# CD38 regulates chronic lymphocytic leukemia proliferation via CD45 phosphatase activity

**DOI:** 10.1016/j.omton.2024.200841

**Published:** 2024-06-24

**Authors:** John F. Imbery, Celina Wiik, Julia Heinzelbecker, Jenny K. Jebsen, Mia K. Dobbing, Nunzio Bottini, Stephanie M. Stanford, Ludvig A. Munthe, Geir E. Tjønnfjord, Anders Tveita, Peter Szodoray, Britt Nakken

**Affiliations:** 1 Department of Immunology, Oslo University Hospital, Oslo, Norway; 2 KG Jebsen Centre for B Cell Malignancies, Institute of Clinical Medicine, Faculty of Medicine, University of Oslo, and Department of Immunology, Oslo University Hospital, Oslo, Norway; 3 Precision Immunotherapy Alliance (PRIMA), Institute of Clinical Medicine, University of Oslo, Oslo, Norway; 4 Kao Autoimmunity Institute and Division of Rheumatology, Cedars-Sinai Medical Center, Los Angeles, CA, USA; 5 Division of Rheumatology, Allergy and Immunology, Department of Medicine, University of California, San Diego, La Jolla, CA, USA; 6 Department of Hematology, Oslo University Hospital, Oslo, Norway

**Keywords:** MT: Regular Issue, CD45, CD38, CLL, Th cells, CD43, galectin-1, chronic lymphocytic leukemia, proliferation, BCR signaling

## Abstract

Chronic lymphocytic leukemia (CLL) growth is dependent on both B cell receptor (BCR) signaling and signals from microenvironmental T helper (Th) cells. We previously described a mechanism where Th cells enhance BCR signaling and proliferation through CD45 phosphatase activity regulation via galectin-1 and CD43. The CLL negative prognostic indicator CD38 is linked to BCR signaling and proliferation, with its expression induced by Th cells. Here, we explore a link between CD38 and CD45 phosphatase activity regulation using patient-derived material in a Th-CLL cell co-culture model. Results demonstrate CD43 and galectin-1 are co-expressed with CD38, defining proliferative CLL cells with augmented CD45 activity. CD38 enzymatic and receptor inhibition regulated CD43 and galectin-1 expression, CD45 activity^hi^ populations, and CLL proliferation, while leaving Th cells largely unaffected. Mechanistically, *CD38*- or *LGALS1* (galectin-1)-deficient malignant B cell lines further confirmed CD38-mediated regulation of CD45 activity and BCR signaling through CD43 expression and galectin-1 surface binding, while galectin-1 contributed to CD43/CD45 colocalization. Together, this highlights CD38 as an important regulator of CD45 activity via CD43 and galectin-1, in turn acting as a positive modulator of CLL proliferation. Ultimately, the CD38/CD45 molecular hub could be an important therapeutic target in CLL.

## Graphical abstract

**Figure:**
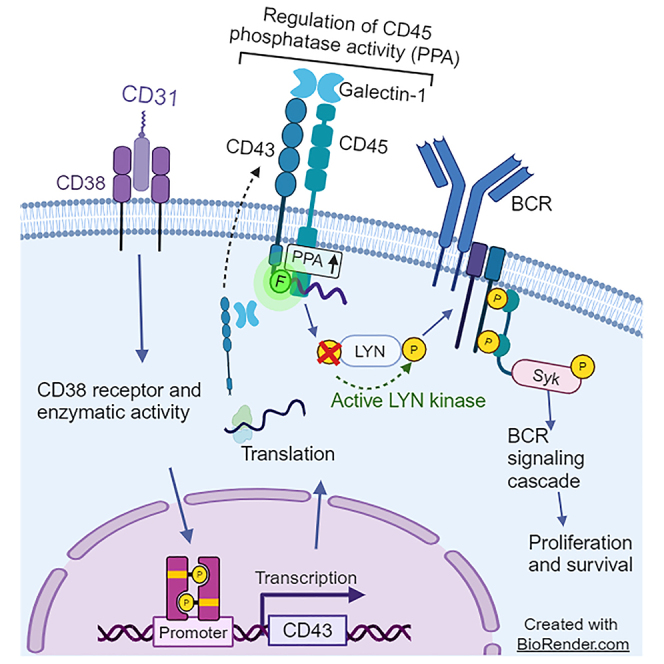

## Teaser

Imbery and colleagues demonstrated that CLL cell proliferation is driven by CD38 regulation of CD45 phosphatase activity via CD43 and galectin-1. Inhibiting CD38 receptor or enzymatic activities reduced CD43 expression and galectin-1 binding, highlighting the potential of targeting the CD38/CD45 molecular hub as a therapeutic strategy in CLL.

## Introduction

Chronic lymphocytic leukemia (CLL) is characterized by clonal expansion of malignant, mature-like CD5^+^ B cells and is the most common adult leukemia in the Western world.^1^ Clinical outcomes are heterogeneous, and several predictors exist that signify a more aggressive CLL disease state. Mutational status of the IgHV gene,^2^ ZAP-70 positivity,^3^^,^^4^ and high CD38 expression^5^^,^^6^^,^^7^^,^^8^ have all been shown to correlate with worse outcomes. Initial reports were inconsistent on whether these indicators were mutually inclusive, but it is now thought that CD38 and IgHV mutational status are independent prognostic factors.^9^^,^^10^

CD38 is a type II transmembrane glycoprotein displaying both NAD^+^ glycohydrolase and ADP-ribosyl cyclase activity, in addition to being able to catalyze a base exchange reaction.^11^ The enzymatic activities of CD38 generate three messengers, with each imparting biological activity by targeting distinct receptors regulating cytosolic calcium levels. Additionally, CD38 can act as receptor to the non-substrate ligand CD31^12^ commonly found on endothelial cells. Stimulation of CD38 receptor function in CLL showed CD38 colocalized with B cell receptor (BCR) signaling components upon cell polarization^13^^,^^14^ and enhanced chemotactic migratory potential of CLL cells.^15^ CD38 receptor function also increased pSyk activation in CLL cells and, when paired with BCR stimulation, synergistically enhanced downstream Erk phosphorylation.^16^ The enzymatic activity of CD38 is also important in CLL for both cell adhesion and *in vivo* homing of the malignant cells.^14^ Previous studies have highlighted the importance of both CD38 enzymatic and receptor function for CLL BCR signaling; however, it is unclear how they mechanistically regulate BCR signaling and proliferation in CLL.

BCR signaling plays a central pathogenic role in CLL,^17^ with its importance underscored by current therapeutics such as Bruton tyrosine kinase inhibitors, which induce remission and improve patient survival.^18^ BCR signaling thresholds are critically regulated by the protein tyrosine phosphatase CD45, which removes an inhibitory phosphate group from Src family kinases,^19^^,^^20^ consequentially potentiating their kinase activity. Since CD45 affects immune cell responses by controlling antigen signaling thresholds, CD45 activity modulation could be a therapeutic target in B cell malignancies.

Microenvironmental factors, particularly T helper (Th) cells, are increasingly acknowledged as essential for the proliferation and survival of CLL cells.^21^^,^^22^ Indeed, CLL cells are capable of presenting antigen and proliferate upon interaction with their cognate Th cell both *in vitro* and *in vivo*.^23^ To reconcile the role of Th cells in CLL proliferation and the CLL cell’s reliance on BCR signaling, we have outlined a mechanism by which Th cells amplify CLL BCR signaling by elevating CD45 activity through the CD45 natural ligand, galectin-1, and the sialoglycoprotein CD43.^24^ This work demonstrated microenvironmental Th cells propagated CLL proliferation by lowering BCR signaling thresholds.^24^ Galectin-1 also enhanced CD45 activity in healthy B cells and we showed this mechanism is crucial for effective differentiation toward antibody-secreting cells in response to T cell help.^25^ In parallel, like CD45 activity, CD38 expression on CLL cells is also regulated by Th cell signals.^26^ Given that both CD45 activity and CD38 exhibit regulatory roles affecting BCR signaling and CLL cell proliferation, we explored a potential link between CD38 and CD45 activity regulation.

Collectively, inhibitory CD38 studies using a patient derived Th-CLL cell co-culture strategy, alongside studies in *CD38-*and *LGALS1* (galectin-1)-deficient malignant B cell lines, showed CD38-mediated CD45 activity regulation via CD43 expression and galectin-1 surface localization, while galectin-1 binding influenced co-localization of CD43 and CD45.

## Results

### CD38 is associated with enhanced CD45 activity, CD43, galectin-1, and proliferation in CLL

**Figure 1:**
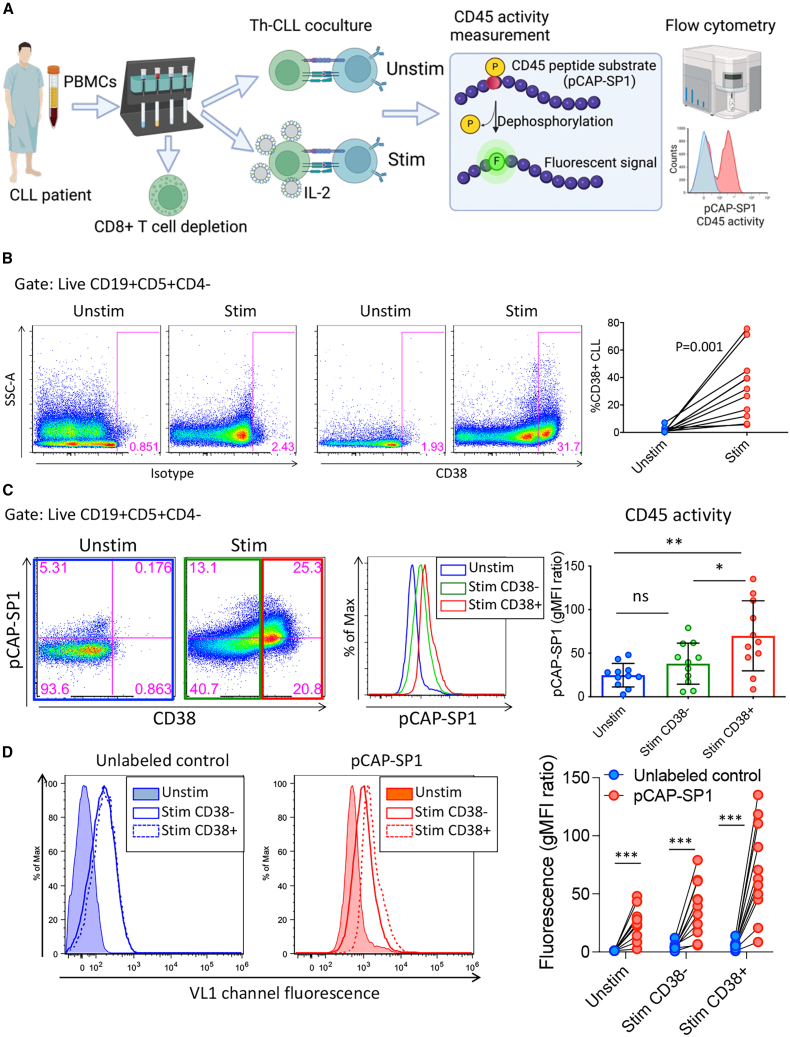
CD38-expressing CLL cells showed enhanced CD45 activity (A) Schematic depicting the Th-CLL cell co-culture experimental setup and CD45 activity measurement. Created with BioRender.com. (B) Stimulated CLL cells increased CD38 expression. The right panels represent flow cytometry scatterplots gated for CD38 positivity in unstimulated and stimulated CLL cells. Left panels are representative scatterplots for the CD38 isotype control from the same cultures. (C) Stimulated CD38^+^ CLL cells had the highest CD45 activity in comparison to stimulated CD38^−^ and unstimulated CLL cells. Representative flow cytometry scatterplots show the association of CD38 expression with CD45 activity. Representative histograms from populations of interest are also included. CD45 activity geometric mean fluorescence intensity (gMFI) was normalized to the gMFI of CLL cells from unstimulated cultures where the CD45 activity probe was omitted (gMFI ratio). (D) No significant changes in autofluorescence were observed in the absence of the CD45 activity probe (pCAP-SP1) in the denoted populations. Included are representative histograms with and without the inclusion of pCAP-SP1. Once again, CD45 activity gMFI was normalized to the gMFI of CLL cells from unstimulated cultures where the CD45 activity probe was omitted (gMFI ratio). Statistical significance was defined with Wilcoxon matched-pairs signed rank test (B), one-way ANOVA with Tukey’s multiple comparisons test (C), and two-way ANOVA with the Geisser-Greenhouse correction and Sidak multiple comparison test (D). Data are representative of 11 CLL patient samples (B–D).

To investigate a connection between CD38 and CD45 activity, we recapitulated the *in vivo* activation of Th cells by increasing the frequency of activated, autologous Th cells via addition of bead-immobilized agonistic CD3/CD28 antibodies and compared this to cultures where Th cells were not activated (Figure 1A). In line with earlier findings, Th cell stimulation of CLL cells upregulated CD38 expression in all patient samples but to variable levels (Figure 1B), reflecting the inherent variability of CD38 expression observed among patients.^26^ To assess CD45 activity in CD38 expressing (and non-expressing) populations, we used a CD45-specific fluorogenic peptide substrate, phosphorylated coumaryl amino propionic acid-SP1 (pCAP-SP1).^24^^,^^25^^,^^27^^,^^28^^,^^29^ pCAP-SP1 is cell permeable and dephosphorylated by active CD45 phosphatase, producing a fluorescent signal that can be captured on flow cytometry (see materials and methods) (Figure 1A). Stimulated CD38^+^ CLL cells showed enhanced CD45 activity compared to stimulated CD38^−^ and overall unstimulated CLL cells (Figure 1C), suggesting a link between CD38 and CD45 activity. As a control, we omitted the CD45 activity probe (pCAP-SP1) and observed minimal autofluorescence within the same populations (Figure 1D).

**Figure 2:**
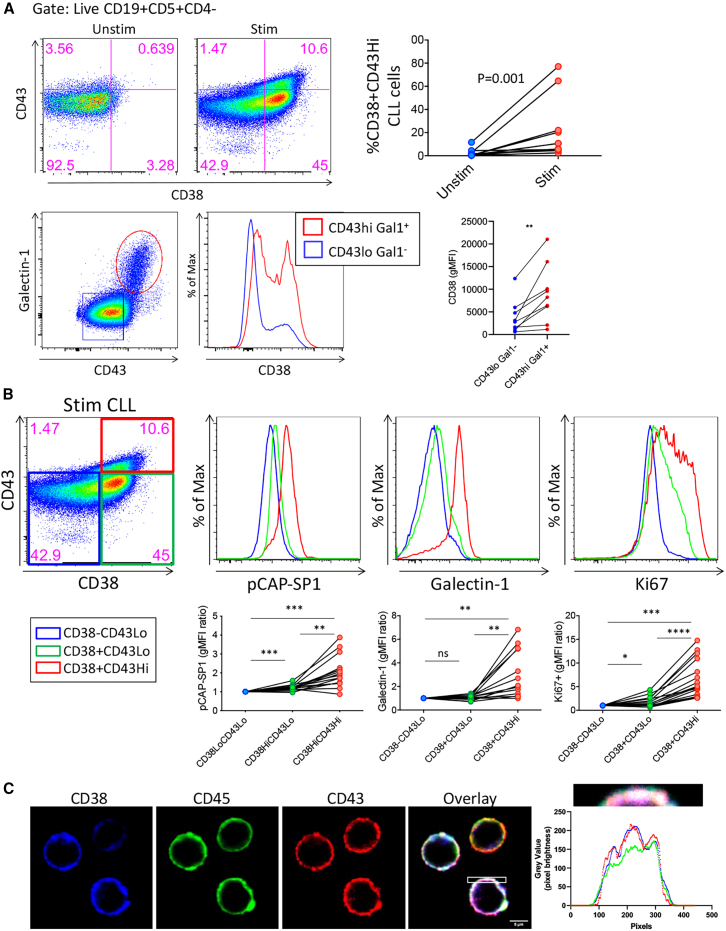
CD38 is co-expressed with known regulators of CD45 activity in activated CLL cells (A) Stimulated CLL cells display a robust increase in the CD38^+^/CD43^hi^ population. Additionally, CD43^hi^/galectin-1^+^ population (a previously identified CD45 activity^hi^ population) has high CD38 expression in comparison to the CD43^lo^/galectin-1^−^ counterpart population. Top row are representative flow cytometry scatterplots of CD38^+^/CD43^hi^ populations in unstimulated and stimulated CLL cells. Bottom row is a representative scatterplot of stimulated CD43^hi^/galectin-1^+^ and CD43^lo^/galectin-1^−^ CLL populations and a representative histogram of CD38 expression in those populations. (B) When assessing stimulated CLL cells, the CD38^+^/CD43^hi^ population had the highest CD45 activity, galectin-1 expression, and proliferative output when compared to CD38^+^/CD43^lo^ and CD38^−^/CD43^lo^ populations. Included are representative histograms for each respective parameter and a representative flow cytometry scatterplot delineating the populations. (C) Confocal images localizing CD38 (blue), CD45 (green), and CD43 (red) in isolated CLL cells. A region of interest was taken from the overlay and the fluorophore intensity (y axis, RGB profiler tool) plotted at a given pixel number (x axis). Statistical significance was defined with Wilcoxon matched-pairs signed rank test (A) and Friedman’s test with Dunn’s multiple comparisons test (B). Data is representative of 11 CLL patient samples (A, top row), 9 patient samples (A, bottom row), and 16 (left), 13 (middle), and 16 (right) patients for (B).

We next characterized CD38-expressing CLL cells by assessing the expression of CD43 and galectin-1, with both previously linked to CD45 activity regulation.^24^^,^^25^ Th cell activation significantly increased a CLL population expressing CD38 and high levels of CD43 (CD38^+^/CD43^hi^), establishing an association between CD38 and CD43 (Figure 2A, top). Conversely, the CD43^hi^/galectin-1^+^ population expressed heightened levels of CD38 (Figure 2A, bottom), linking CD38 expression to a population harboring high CD45 activity and CLL proliferative capacity.^24^ Dividing CLL cells based on CD38 and CD43 expression (CD38^−^/CD43^lo^, CD38^+^/CD43^lo^, CD38^+^/CD43^hi^) showed the combination of CD38^+^/CD43^hi^ associated with enhanced proliferation, galectin-1 expression, and CD45 activity in CLL (Figure 2B). Findings were extended by surface localization of CD45, CD43, and CD38 using confocal microscopy on purified, unstimulated CLL cells where CD38 expression was induced by supplementing pure CLL cultures with interferon gamma. Images reveal a tripartite co-localization of CD45, CD43, and CD38 on CLL cells (Figure 2C), in addition to overlapping areas of high pixel intensity in three-dimensional surface plots (Figure S1), suggesting the existence of a molecular hub comprising these proteins.

CD38-expressing CLL cells exhibited enhanced CD45 activity, with CD43 and galectin-1 co-expressed alongside CD38, suggesting CD38 may control high CD45 activity in CLL cells.

### CD38 enzymatic inhibition decreases CLL proliferation and CD45 activity^hi^ populations

**Figure 3:**
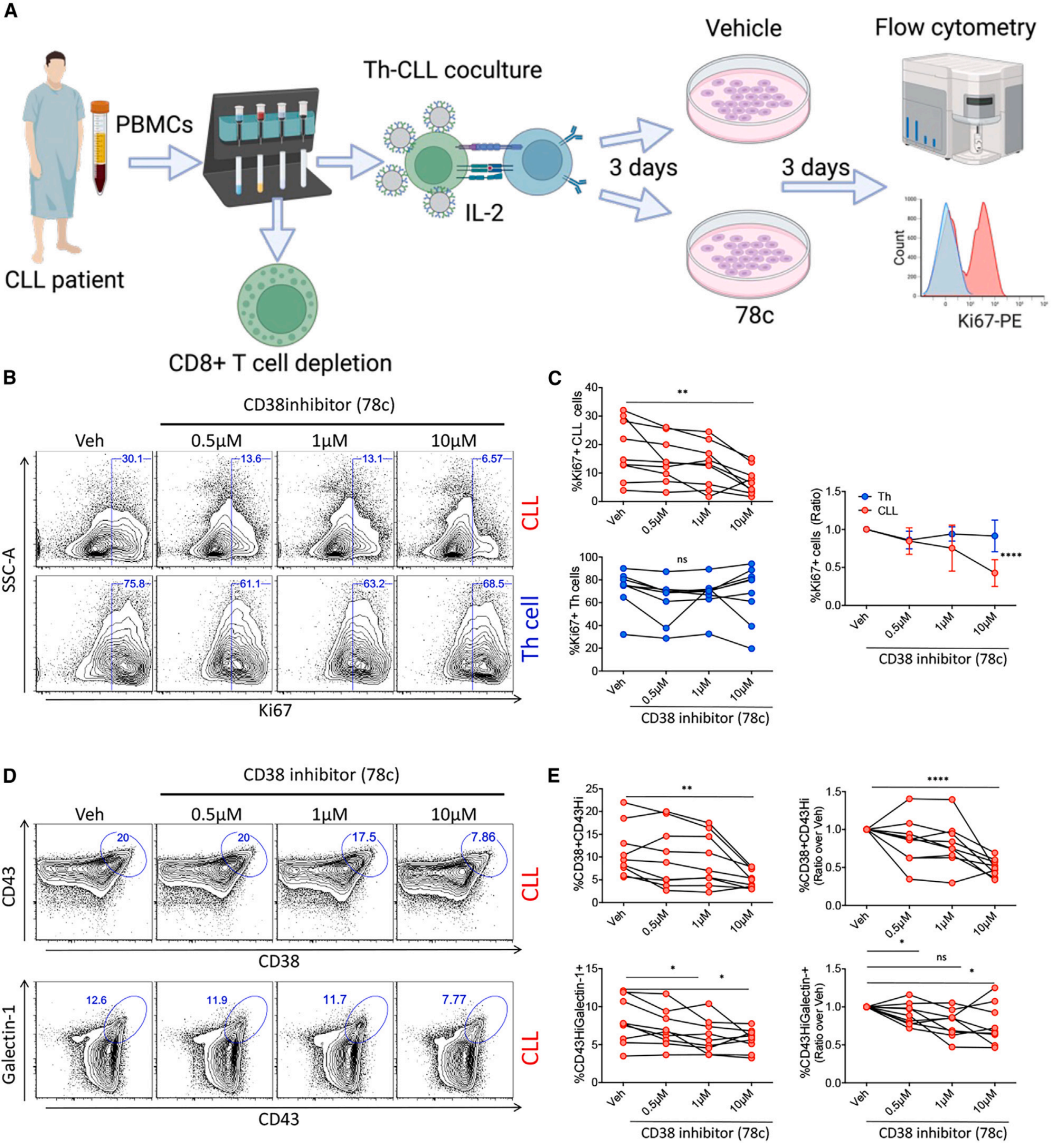
Inhibiting CD38 enzymatic activity reduced CLL proliferation and CD45 activity^hi^ populations (A) Schematic depicting the experimental setup. Created with BioRender.com. (B and C) Treatment with the CD38 enzymatic inhibitor 78c in Th-CLL cell co-cultures reduced the CLL proliferative (Ki67^+^) output in a concentration-dependent manner. Representative flow cytometry scatterplots of CLL (top row) and Th cell (bottom row) proliferation at the indicated concentrations. Th cell proliferation remained unaffected. %Ki67^+^ was normalized as ratio over vehicle for Th versus CLL comparison. (D and E) Treatment with 78c also selectively decrease the CD45 activity^hi^ populations CD38^+^/CD43^hi^ (top row) and CD43^hi^/galectin-1^+^ (bottom row) in CLL cells. Included are representative flow cytometry scatterplots. Normalized graphs (ratio over vehicle) have also been provided for comparison. Statistical significance was calculated by Friedman’s test with Dunn’s multiple comparisons test (C), repeated-measures one-way ANOVA with Dunnett’s multiple comparisons test (E), and two-way ANOVA for Th versus CLL cell comparisons. Data are representative of eight CLL patient samples.

The initial experiments revealed an association between CD45 activity and CD38 in CLL. To further a connection between CD45 activity and CD38, we interrogated the functional consequences of inhibiting CD38 enzymatic activity using the small molecule inhibitor 78c^30^^,^^31^ in the Th-CLL cell co-culture model (Figure 3A). Assessment of the proliferative CLL signature using Ki67 demonstrated a significant decrease in CLL proliferation upon inhibition of CD38 enzymatic activity (Figures 3B and 3C). Noticeably, the proliferative output of activated Th cells from the same co-cultures, which also express CD38, was unaffected (Figures 3B and 3C). This suggests CD38 enzymatic activity is a more important driver of CLL proliferation in this experimental model. Strikingly, the use of 78c led to a selective decrease in CD38^+^/CD43^hi^ and CD43^hi^/galectin-1^+^ CLL populations upon increasing concentrations of inhibitor (Figures 3D and 3E), indicative of CD45 activity regulation by CD38.

Furthermore, CD38 enzymatic inhibition decreased CD43 protein expression in both CLL and Th cells (Figure S2A), alongside a moderate decrease in CD38 geometric mean fluorescence intensity (gMFI) in CLL cells with unchanged Syk kinase activation (pSyk) (Figures S2C and S2D). Together with unaffected cell viability (Figures S3A and S3B), these data are suggestive of 78c-mediated inhibition of CLL proliferation via CD38 and CD43.

CD38 enzymatic inhibition selectively reduced CLL proliferative output and down-modulated CD43, CD38, and CD45 activity^hi^ populations.

### Inhibition of CD38 receptor function decreases CLL proliferation and CD45 activity^hi^ populations

**Figure 4:**
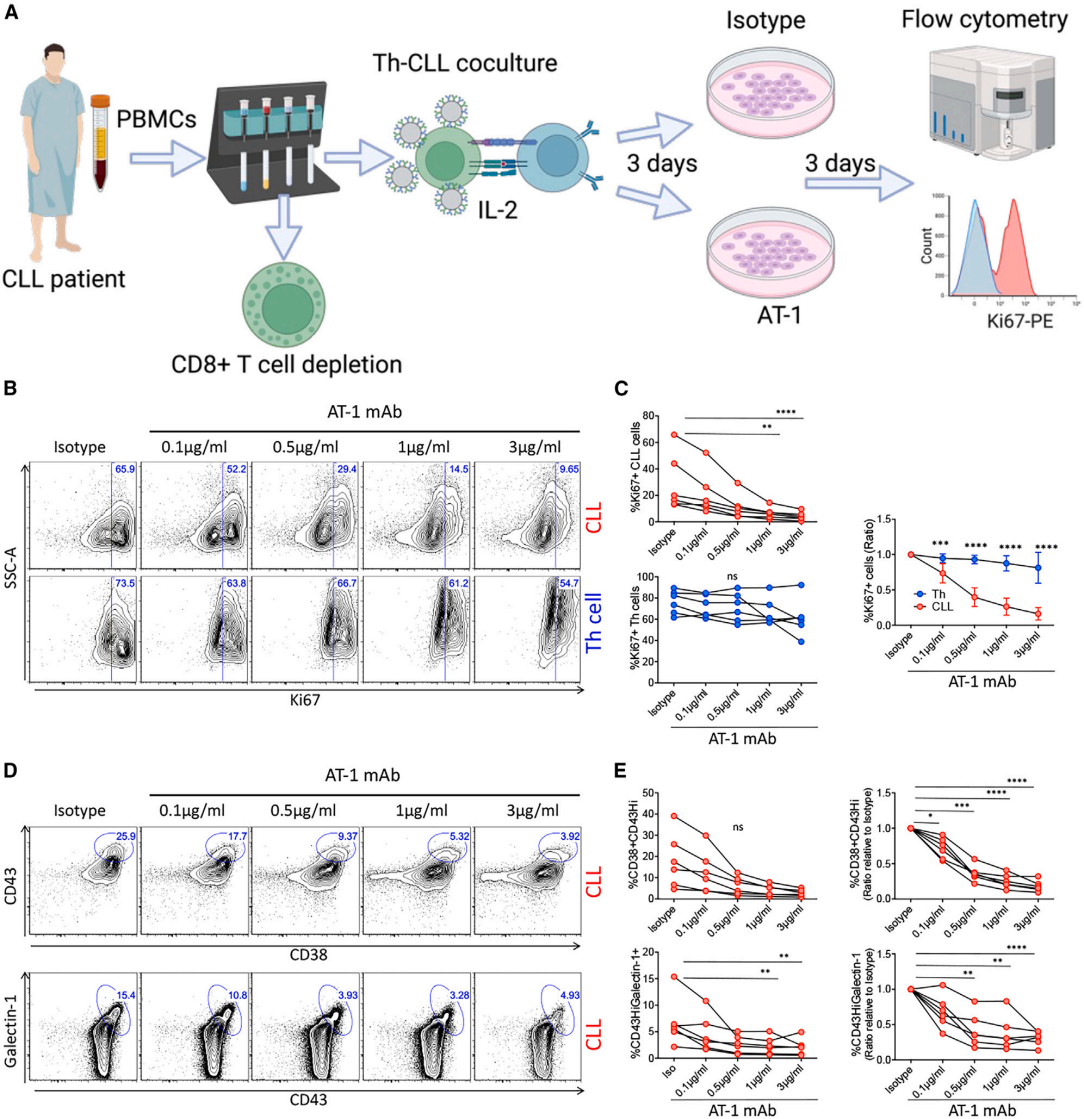
The CD38 blocking mAb AT-1 reduced CLL proliferation and CD45 activity^hi^ populations (A) Schematic depicting the experimental setup. Created with BioRender.com. (B and C) Treatment with increasing concentrations of the mAb AT-1 reduced CLL cell proliferation (Ki67^+^) while leaving Th cell proliferation largely intact. Included are representative flow cytometry scatterplots of CLL (top row) and Th cells (bottom row). %Ki67^+^ was normalized as ratio over isotype for comparative graphs. (D and E) Increasing concentration of the mAb AT-1 selectively decreased the CD45 activity^hi^ populations CD38^+^/CD43^hi^ (top row) and CD43^hi^/galectin-1^+^ (bottom row). Representative flow cytometry scatterplots from the indicated concentrations are included. To account for the inherent variability of the CD45acivity^hi^ population between CLL patients (left graphs), data was normalized to isotype control values (right graphs). Statistical significance was defined by Friedman’s test with Dunn’s multiple comparisons test (C and E [CLL CD43^hi^/galectin-1^+^ left graph]), repeated-measures one-way ANOVA with Dunnett’s multiple comparisons test (rest of E), and two-way ANOVA for Th versus CLL cell comparisons. Data are representative of six CLL patient samples.

As it is unclear whether the enzyme and receptor properties of CD38 have similar functional consequences, we also interrogated the outcomes of CD38 receptor inhibition by using a monoclonal CD38 blocking antibody (AT-1)^15^ administered to Th-CLL cell co-cultures (Figure 4A). We first explored expression of the CD38 ligand CD31 and found constitutive expression in both CLL and Th cells (Figures S4A–S4C), with higher CD31 expression in CLL cells compared to Th cells (Figure S4D), confirming the presence of an agonist for CD38 receptor activation. Treatment with AT-1 monoclonal antibody (mAb) produced a concentration-dependent decrease in CLL proliferation while leaving the Th cell Ki67^+^ population largely intact (Figures 4B and 4C). Analogous to enzymatic inhibition, a decrease in proliferation was accompanied by a decrease in CD38^+^/CD43^hi^ and CD43^hi^/galectin-1^+^ CLL cells (with high CD45 activity) when normalized to account for high patient variability in these populations (Figures 4D and 4E). In line with this, receptor block also produced a significant reduction in both CLL and Th cell CD43 expression (Figure S5A), followed by a selective decrease in CLL galectin-1 expression (Figure S5B), which may account for the specific inhibition of proliferating CLL cells. There was also a decreasing trend in overall CD38 protein expression on CLL cells (Figure S5D) that was not attributable to competition of AT-1 with the CD38 antibody used for flow cytometry (Figure S6A). Since overall CLL and Th cell viability was unchanged (Figures S6B and S6C), data are suggestive of gene regulation effects or receptor-mediated endocytosis.

Strikingly, activation of pSyk in CLL cells was reduced upon use of mAb AT-1, while levels in Th cells remained low (Figure S5C). Use of pSyk inhibitor R406 confirmed that active Syk kinase plays an important role in Th-mediated CLL proliferation by decreasing CD45 activity^hi^ populations, potentially via CD43 regulation (Figures S7–S9). This suggests a role for CD38 receptor function in modulation of CLL pSyk activation, a result not observed upon CD38 enzymatic inhibition.

For comparison, we tested the clinically relevant human CD38 mAb daratumumab (DARA), which is a weak inhibitor of CD38 enzymatic activities.^32^ In our experimental model, the data revealed only a modest effect on CLL cell proliferation (Figure S10A), but with significant death of CD38^+^ CLL and Th cells (Figures S10B and S10C). In contrast with AT-1, DARA did not affect pSyk activation (Figure S10D), further indicating a different mechanism of action. Interestingly, the CD43^hi^/galectin-1^+^ CLL population showed only modest reduction upon DARA treatment compared to overall CD38^+^ CLL decrease (Figure S10E). Of note, we used anti-human IgG antibody to reveal DARA binding at a low concentration did not inhibit binding of the CD38 flow antibody (Figure S10F). We found that DARA may bind the proliferative CD43^hi^/galectin-1^+^ population to a lesser extent than overall CD38^+^ CLL cells (Figure S10G). This could indicate that CD43 and/or galectin-1 is masking the DARA epitope on CD38 causing proliferating CLL cells to evade DARA treatment.

Collectively, blocking CD38 receptor function with AT-1 selectively diminished CLL proliferation and pSyk activation along with CD45activty^hi^ CLL populations, CD43, galectin-1, and CD38 expression, while DARA broadly targeted CD38^+^ cells.

### CLL-intrinsic effect of CD38 enzymatic and receptor inhibition

**Figure 5:**
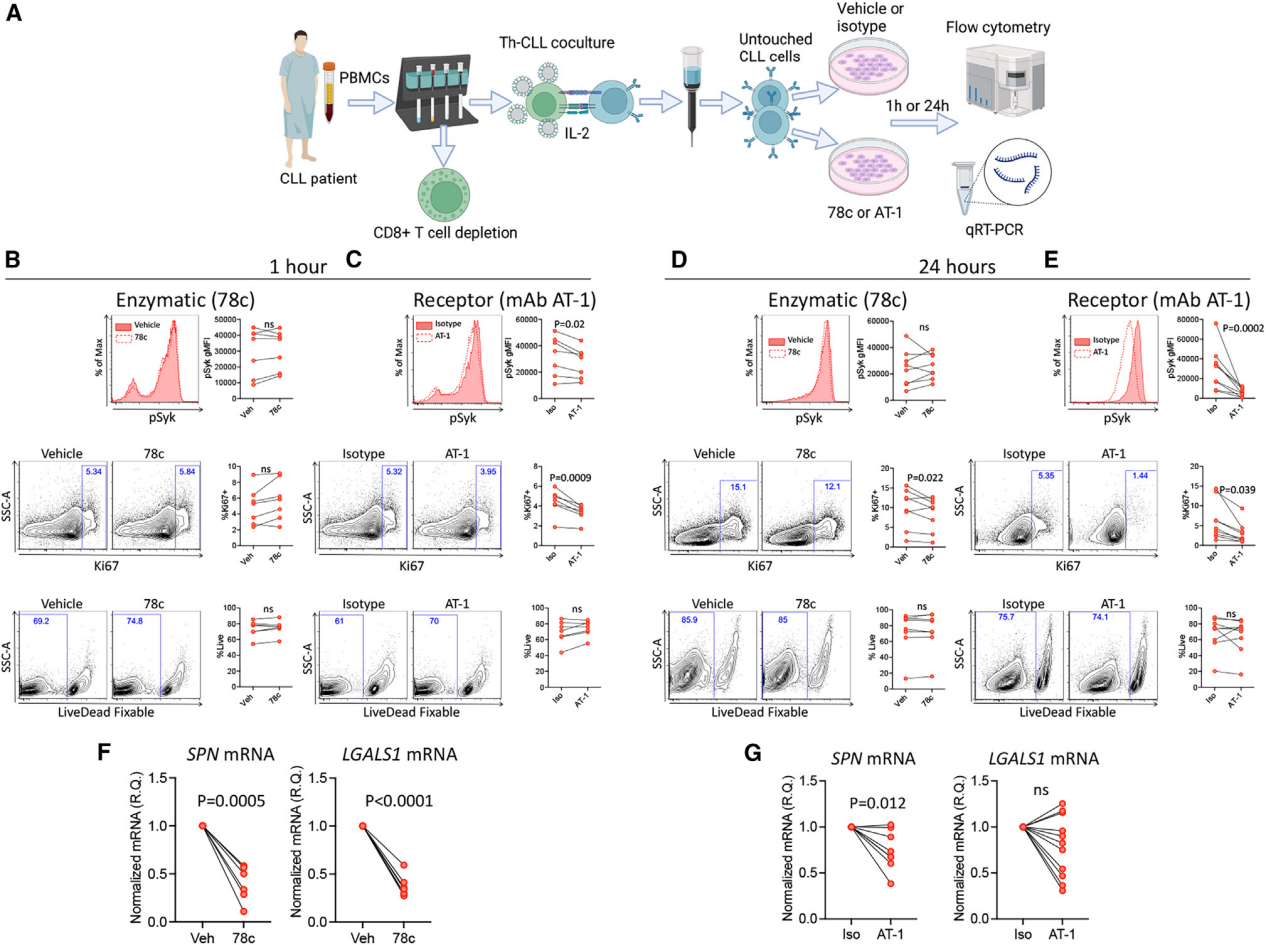
CLL-intrinsic effect of CD38 enzymatic and receptor inhibition (A) Schematic depicting the experimental setup. Created with BioRender.com. Isolated CLL cells were treated for 1 or 24 h with CD38 enzymatic inhibitor 78c (B and D) or mAb AT-1 (C and E). One-hour inhibition with 78c did not alter CLL pSyk activation, proliferation, or cell viability (B). Conversely, 1-h inhibition with AT-1 reduced CLL pSyk activation and proliferation, while similarly leaving cell viability unaffected (C). Included are representative histograms for pSyk and representative flow cytometry scatterplots. The 24-h enzymatic inhibition did not affect pSyk activation or cell viability, but decreased the proliferative output of CLL cells (D). Alongside a concomitant reduction in proliferation without reduction in cell viability, 24-h receptor block robustly reduced CLL pSyk activation (E). Examination of *SPN* (CD43) and *LGALS1* (galectin-1) gene expression at 24 h 78c treatment revealed a robust decrease in both *SPN* and LGALS1 mRNA levels (F), while AT-1 treatment revealed a significant reduction in *SPN* mRNA with a near significant reduction in *LGALS1* mRNA (*p* = 0.065) (G). All mRNA expression levels are relative quantity normalized to *POLR2A* (F and G). Statistical significance was defined with Student’s ratio paired t test (B–D [pSyk, Ki67], E [pSyk]), Wilcoxon matched-pairs signed rank test (D [live/dead], E [Ki67, live/dead]), and Student’s paired t test (F and G). Data are representative of 6 (F left graph), 7 (B, C, F right graph), 8 (D, G left graph), 9 (E), and 11 (G right graph) CLL patient samples.

As activated Th cells also express CD38, the effects observed on CLL cells from co-culture experiments where CD38 enzymatic or receptor activity was inhibited may be an indirect effect mediated by modulation of Th cell signaling. To explore if our data are reflective of CLL-intrinsic effects, we negatively isolated CLL cells following co-culture with activated Th cells (Figure 5A). Purified CLL cells were given 78c (enzymatic inhibition) or AT-1 (receptor inhibition) and harvested following a 1- or 24-h incubation period (Figure 5A).

Given uncertainties regarding the permeability and pharmacokinetics of 78c, 1-h experiments used a supramaximal concentration (100 μM). Even at such a high concentration, CD38 enzymatic inhibition did not result in modulation of pSyk activation, proliferation (Ki67^+^) or cell viability (Figure 5B). However, CLL cells treated with the CD38-blocking mAb AT-1 produced a noticeable decrease in pSyk activation after 1-h, which was coupled to decrease in the proliferative (Ki67^+^) output of CLL cells (Figure 5C). Overall cell viability with CD38 AT-1 was unaffected (Figure 5C), meaning observed changes may result from changes to downstream signaling pathways. Ultimately, these experiments revealed rapid CLL-intrinsic down-modulation of proliferation upon receptor, but not enzymatic, inhibition, suggesting the likelihood of different mechanisms of action.

Analysis following 24-h of 78c treatment demonstrated CLL cells maintained a similar pSyk activation level, yet there was a signification decrease in the proliferative output without a concomitant effect on CLL cell viability (Figure 5D). To mechanistically link CD38 enzymatic activity to CD45 activity regulation, we quantified expression of CD45 activity regulators *SPN* (encoding CD43) and *LGALS1* (encoding galectin-1) using quantitative real-time PCR. CD38 inhibition with 78c produced a robust decrease in the gene expression of *SPN* and *LGALS1* (Figure 5F), strengthening the notion that down-modulation of CD45 activity occurs through CD38 enzymatic regulation of CD43 and galectin-1.

Comparatively, results from 24-h AT-1 experiments largely mimicked those seen after 1-h, i.e., decreasing pSyk activation and CLL proliferation without affecting overall CLL cell viability (Figure 5E). To determine if CD38 receptor block may also be connected to CD45 activity regulation, we assessed *SPN* and *LGALS1* mRNA levels. Results revealed a decrease in *SPN* gene expression with a trend toward reduction in *LGALS1* (Figure 5G), suggestive of CD45 activity modulation via CD38 receptor function.

Taken together, CD38 enzymatic and receptor inhibition reduced CLL cell proliferation and mRNA expression of CD45 activity regulators CD43 and galectin-1. There was an additional reduction in pSyk activation observed upon receptor inhibition, furthering support of overlapping yet unique CD38 signaling pathways.

### CD38 regulates BCR signaling and CD45 activity through CD43 and galectin-1 in malignant B cells

**Figure 6:**
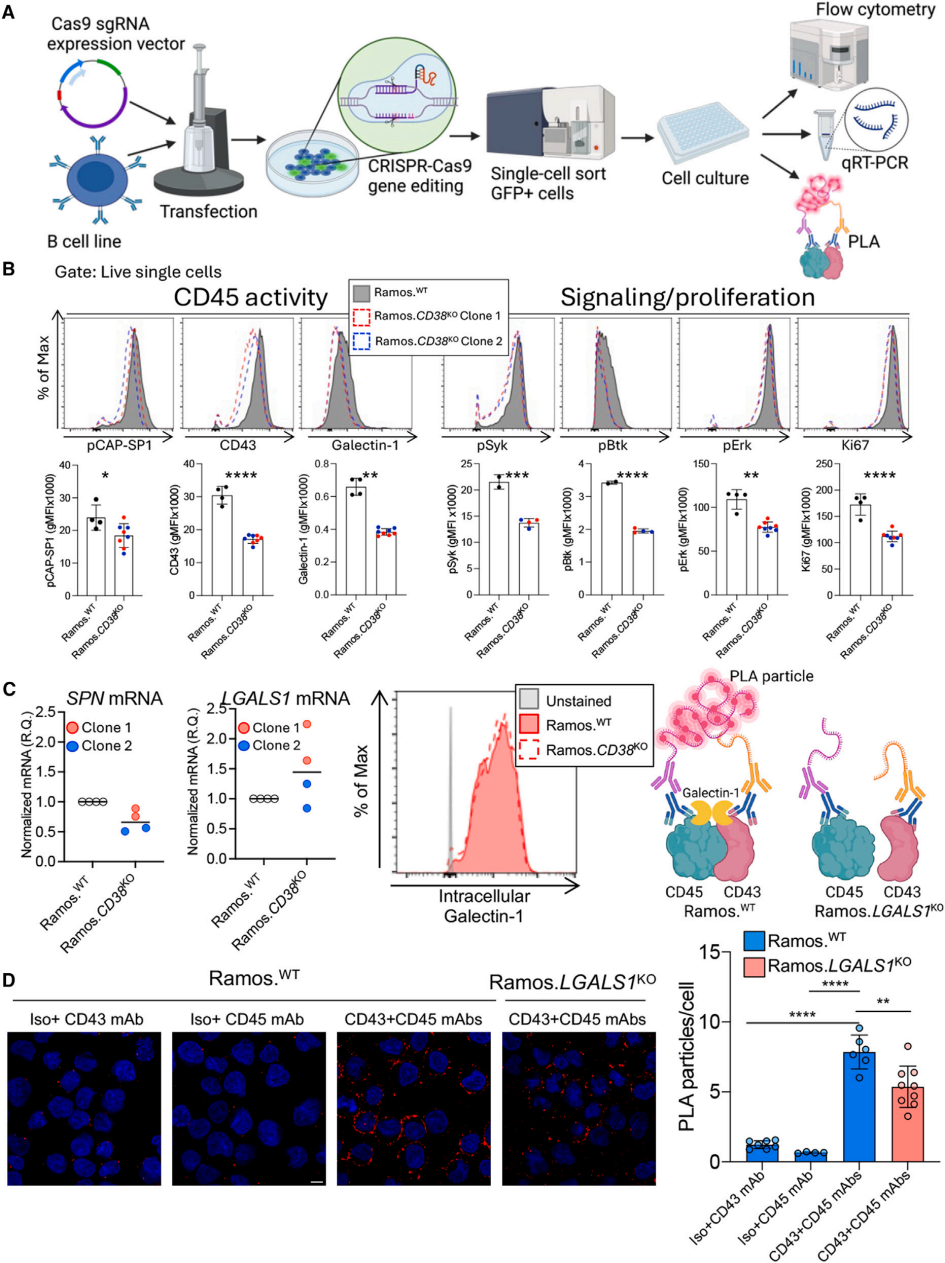
CD38 regulates BCR signaling and CD45 activity through CD43 and galectin-1 in malignant B cells (A) Schematic depicting generation of CRISPR-Cas9 gene edited cells and the downstream workflow. Created with BioRender.com. (B) When comparing to Ramos.^WT^, Ramos.*CD38*^KO^ cells demonstrated reduced surface expression of CD43 and galectin-1, alongside a concomitant reduction in CD45 activity (pCAP-SP1). Included are representative histograms (Ramos.^WT^ filled histograms, Ramos.*CD38*^KO^ dashed histograms). Investigation of proximal (pSyk, pBtk) and distal (pErk) BCR signaling kinases showed decreased activation of all three in Ramos.*CD38*^KO^ cells. Included are representative histograms (Ramos.^WT^ filled histograms, Ramos.*CD38*^KO^ dashed histograms). Data were obtained from two individual Ramos.*CD38*^KO^ clones (A and B). (C) On the mRNA level, *SPN* (CD43) gene expression was reduced in Ramos.*CD38*^KO^ cells while LGALS1 (galectin-1) gene expression was not. Intracellular galectin-1 expression was consistent between Ramos.^WT^ and Ramos.*CD38*^KO^ cells. Included are representative histograms. (D) Confocal images with the red signal representing co-localization of CD43/CD45 (PLA particles, see schematic created with BioRender.com.; scale bar, 5 μm). Analysis revealed the PLA particles were significantly reduced in Ramos. *LGALS1*^KO^ cells in comparison to Ramos.^WT^. Statistical significance was determined by Student’s unpaired t test (B: pCAP-SP1, CD43, pSyk, pBTK, Ki67), Mann-Whitney Test (B: pErk, galectin-1), and one-way ANOVA with Tukey’s multiple comparisons test (D).

To further confirm CD38-mediated regulation of CD45 activity, we performed genetic ablation of *CD38* and *LGALS1* (galectin-1). Currently available cell lines originating from CLL patients MEC1^33^ and MEC2,^33^ PCL12,^34^ OSU-CLL,^35^ and MDA-BM5^36^ may represent EBV^+^ B lymphoblastoid cells and are thus poor models for CLL.^37^ In addition to the more progressed leukemic phenotype, most of these CLL cell lines have reduced CD38-expression,^33^^,^^34^^,^^35^^,^^36^ therefore we utilized the mature B lymphoma-derived cell line, Ramos, expressing high levels of CD38^38^ and generated knockout variants (Ramos.*CD38*^KO^ and Ramos.*LGALS1*^KO^) using CRISPR-Cas9 (Figures 6A, S11A, and S11B). Loss of CD38 expression resulted in decreased surface expression of CD43 and galectin-1 followed by diminished CD45 activity (Figure 6B). This is consistent with CD38-mediated modulation of CD45 activity through CD43 and galectin-1 regulation. Given the decrease in CD45 activity and its role in antigen signaling and proliferation, we investigated downstream BCR signaling kinases pSyk, pBtk, and pErk, as well as Ki67. Both proximal and distal BCR signaling kinase activation and Ki67 expression were reduced in Ramos.*CD38*^KO^ cells (Figure 6B), in line with CD38-mediated regulation of BCR signaling and proliferation via CD45 activity.

Quantitative real-time PCR evaluation revealed decreased *SPN* (CD43) but not *LGALS1* (galectin-1) mRNA levels in Ramos.*CD38*^KO^ cells (Figure 6C), confirming regulation of *SPN* gene expression by CD38. We next investigated intracellular galectin-1 protein expression and detected equal levels between Ramos.^WT^ and Ramos.*CD38*^KO^ (Figure 6C), suggesting that CD38 likely regulates CD43 expression, which then influences galectin-1 surface binding.

We previously showed that removal of *LGALS1* by CRISPR-Cas9 in the Raji cell line resulted in diminished CD45 activity^25^ and BCR signaling.^24^ To elucidate the effect of galectin-1 surface binding, we utilized a proximity ligation assay (PLA) to detect colocalization between CD45 and CD43 in the Ramos.*LGALS1*^KO^ cell line. PLA images show reduced colocalization of CD45 and CD43 in Ramos.*LGALS1*^KO^ compared to Ramos.^WT^ (Figure 6D), suggesting that galectin-1 contributes to CD45/CD43 bridging. Moreover, Ramos.*LGALS1*^KO^ cells exhibited reduced CD43 protein and mRNA expression (Figures S11B and S11C), demonstrating that galectin-1 also contributes to CD43 expression. This supports CD38-mediated regulation of CD45 phosphatase activity through CD43 expression and galectin-1 surface binding, where galectin-1 contributes to surface localization of CD43 with CD45.

These results confirm that CD38 controls CD45 activity via regulation of CD43/galectin-1, leading to decreased downstream BCR signaling and proliferation.

## Discussion

Continually evolving research in CLL has led to improved patient outcomes and characterization of prognostic indicators that help to identify at-risk patient subgroups, yet CLL remains classified as incurable. This emphasizes a persistent need for a better understanding of CLL survival signals; therefore, CD38 has steadily gained traction as a protein of interest given its prognostic significance. Here, we used a patient-derived co-culture model together with mechanistic studies in CRISPR-Cas9 gene edited malignant B cell lines to show that CD38 regulated CD43 and galectin-1 expression. This subsequently controlled CD45 activity, BCR signaling, and CLL proliferation. Importantly, these results indicate a therapeutic potential for targeting the CD38/CD45 molecular hub in CLL.

Evidence provided here supports the idea that CD38 contributes to CLL pathogenesis, rather than being a passive activation marker. Inhibition of enzymatic activity decreased CLL proliferation in a CLL-intrinsic manner. This is in line with an earlier report linking CD38 enzymatic activity to CLL cell growth,^14^ while also providing mechanistic insight through selectively down-modulating CD45 activity^hi^ subpopulations. A pertinent question is then which messenger synthesized by CD38 enzymatic activity is central to driving CLL cell signaling? The small molecule inhibitor 78c is a 10 times more potent inhibitor of CD38 hydrolase activity (ADPR) than its corresponding cyclase activity,^31^ with its effect on NAADP synthesis unknown. This would suggest the potential for ADPR as a central player in driving CLL cell function. A thorough investigation using selective inhibitors is certainly warranted to assess each messenger’s individual contribution to CLL cell signaling.

Inhibition of CD38 receptor function with the blocking mAb AT-1 produced remarkably similar results to 78c enzymatic inhibitor experiments. This included selective down-modulation of CLL CD45 activity^hi^ populations and proliferation both during Th CLL co-culture and in a CLL-intrinsic manner. The CLL-intrinsic effect observed using mAb AT-1 also suggests that CD31 present on CLL cells is capable of ligating CD38. Additionally, results inhibiting either CD38 enzymatic or receptor function yielded remarkable similar downstream effects, but with two clear mechanistic distinctions: (1) use of mAb AT-1 robustly reduced pSyk activation and (2) effects of mAb AT-1 were immediate (albeit modest), observable after 1-h. The decreases in pSyk activation by CD38 receptor inhibition are consistent with previous studies where CD31 stimulation increased pSyk levels in CLL cells^16^; conversely, DARA treatment decreased pSyk activation upon BCR stimulation.^39^ Intriguingly, CD38 is capable of polarizing with the BCR complex in malignant B cells and associates with Syk upon anti-IgM crosslinking in healthy B cells.^40^ This fits a model where triggering of CD38 receptor function leads to immediate lateral associations with proteins in the CLL membrane/BCR complex to drive pSyk activation, while downstream CD38 signaling would be responsible for gene regulatory effects (e.g., *SPN* and *LGALS1*). Conversely, triggering of CD38 enzymatic function leads to slower mobilization of cytosolic calcium, which may be restricted both spatially and temporally and does not drive immediate proximal pSyk activation via re-arrangement of the BCR complex. One report demonstrated a decrease in pSyk upon CD38 enzymatic inhibition.^39^ However, this study (1) investigated pSyk upon BCR crosslinking, rather than the tonic signaling addressed in our experiments and (2) used the small molecular inhibitor kuromanin, a flavonoid, that may have different pharmacokinetics and biological properties than 78c. Ultimately, our data fit a model where enzymatic and receptor functions are dual on-going processes eliciting distinct signaling pathways that may converge downstream to regulate transcriptional output, in our case *SPN* and *LGALS1*, important for CD45 activity.

CD38 is an increasingly important prognostic marker in B cell malignancies. Outside of CLL, studies in mantle-cell lymphoma,^41^ diffuse large B cell lymphoma,^42^ and B cell acute lymphoblastic leukemia^43^ correlated CD38 expression to adverse survival rates, while CD38 is constitutively expressed on multiple myeloma cells.^44^ CD38’s prognostic significance created therapeutic interest and the mAb CD38 antibody DARA is approved for use as a combinatorial treatment in multiple myeloma.^45^^,^^46^^,^^47^ This clinical progress spurred DARA research in CLL, where it was found to both kill and inhibit migration *in vitro* and *in vivo,* mainly by immune-effector mechanisms.^48^ In addition, DARA decreased acute BCR signaling,^39^ and synergized with ibrutinib to inhibit tumor growth *in vivo*.^39^ In agreement with these studies, we found that DARA worked mainly by reducing the amount of live CD38^+^ Th and CLL cells. Complement-dependent cytotoxicity is a weak inducer of CLL cell death^48^ and in our experimental system complement was inactivated. Thus, it is likely that DARA, in our experimental setting, induced cell death via immune-effector mechanisms such as antibody-dependent cell-mediated cytotoxicity.^49^^,^^50^ Although we observed that DARA blocked binding of the CD38 flow cytometry antibody, a robust effect was already observable at a low DARA concentration where binding of our CD38 flow antibody was not inhibited. Additionally, the proliferating CD43^hi^/galectin-1^+^ CLL population was only modestly targeted upon DARA treatment. At the same low DARA concentration, we observed less DARA binding to proliferating CLL cells (CD43^hi^/galectin-1^+^) than in overall CD38^+^ cells. Thus, it is tempting to speculate that CD43 and/or galectin-1 is masking the DARA epitope on CD38 causing proliferating CLL cells to evade DARA treatment.

The important role of galectin-1 is highlighted by our previous studies in a *LGALS1*-deficient malignant B cell line, where we demonstrated regulation of both CD45 activity^25^ and BCR signaling.^24^ Moreover, we previously showed that removing surface galectin-1 with the inhibitor OTX008 rendered activated Th cells susceptible to cell death while diminishing CLL proliferation.^24^ Analogously, targeting CD43 resulted in down-modulation of CD43 in both CLL and Th cells, followed by selective reduction of surface galectin-1 and proliferation in CLL cells.^24^ This emphasizes the importance of galectin-1 in CLL proliferation and may suggest that galectin-1 depends on CD43 for surface binding in CLL, but not Th cells, explaining the observed differences in CD38 and CD43 targeting on CLL and Th cell proliferation. CD38 enzymatic inhibition down-regulated both *SPN* and *LGALS1* in CLL cells, in line with a requirement for both CD43 and galectin-1 in CLL proliferation. However, CD38 receptor block resulted in down-modulation of CD43 in both CLL and Th cells, which was coupled to a selective reduction of surface galectin-1, which may account for the specific inhibition of proliferating CLL cells while leaving Th cells unaffected. Equally feasible could be that stimulation via CD3/CD28 activating beads may override any CD38 inhibitory effects on Th cells.

In support of our hypothesis, mechanistic studies in knockout cell lines demonstrated CD38-mediated regulation of CD45 activity by CD43 expression and galectin-1 surface binding, while galectin-1 co-localized CD43 and CD45, supporting galectin-1 and CD43 interaction in B cells. The importance of galectin-1 in localization of surface receptors is an increasingly common theme in the literature.^51^^,^^52^^,^^53^^,^^54^ In fact, for dendritic cells, galectin-1 was shown to co-cluster CD43/CD45 resulting in activation and migration through Syk and protein kinase C signaling.^54^ Our data is in line with a role for CD38 in controlling CD43 expression, which facilitates galectin-1 surface binding and consequentially co-clustering of CD43/CD45.

In summary, CD38 regulated CD45 activity, BCR signaling, and proliferation through CD43 and galectin-1 modulation. Future studies using antibody design around AT-1 or anti-CD43 could provide novel therapeutic strategies targeting proliferating CLL cells while leaving the Th cell compartment intact and thus retain valuable adaptive immunity. Ultimately, the CD38/CD45 molecular hub could be an important therapeutic target in CLL.

## Materials and methods

### Patient samples and peripheral blood mononuclear cell isolation

Patients diagnosed with CLL (Table S1) were recruited through the hematological outpatient clinics at Department of Haematology, Oslo University Hospital, Norway, and peripheral blood samples procured following informed consent. All ethical approvals were granted by the Regional Ethics Committee for Medical and Health Research Ethics (approval number 2016-947, 2016-1466). Samples were subsequently gradient centrifuged (LymphoPrep, Alere Technologies) and peripheral blood mononuclear cells (PBMCs) placed into culture media (RPMI-1640 supplemented with 10% FCS, 1 mM sodium pyruvate, 1× non-essential amino acids, 50 nM of the antioxidant 1-thiglycerol, and 12 μg/mL of the antibiotic gentamicin) or cryopreserved for later use.

### Cell culture

#### Co-culture: Th and CLL cells

CLL PBMCs were first depleted of CD8^+^ cytotoxic T cells using the Dynabeads CD8 Positive Isolation Kit (Thermo Fisher Scientific) and divided into two groups. In group 1, Th cells were given Dynabeads Human T-Activator CD3/CD28 beads (Thermo Fisher Scientific) in combination with IL-2 (20 U/mL) to increase the frequency of activated Th cells. Hereafter, this will be known as stimulated CLL. Conversely, in group 2 the Th cells were not activated and are referenced as unstimulated CLL throughout the remainder of the paper. For an illustration, please see Figure 1A. Co-culture proceeded for 72 h. CD38 enzymatic inhibitor (78c, Tocris), monoclonal CD38 antibody (AT-1, Santa Cruz Biotechnology), or vehicle/isotype antibody control were added at the given concentrations (see text) after 72 h and incubated for an additional 72 h prior to cell harvesting.

#### CLL cell culture

CLL cells were negatively isolated from stimulated Th-CLL cell co-cultures after 72 h with the B-CLL Cell Isolation Kit (Miltenyi Biotec). Isolated CLL cells were cultured either with the CD38 enzymatic inhibitor 78c (or vehicle control) or the CD38 blocking antibody AT-1 (or isotype antibody control) for 24 h before cells were harvested and subjected to flow cytometry or quantitative real-time PCR analyses.

### Flow cytometry-based CD45 activity assay

A method to quantitate CD45 phosphatase activity at the single-cell level using a novel, fluorogenic CD45 peptide probe is previously described^28^ and has been used extensively in our previous projects.^24^^,^^25^^,^^27^^,^^29^ Briefly, a phosphotyrosine mimic, phosphorylated coumaryl amino propionic acid (pCAP), was incorporated into a cell-permeable peptide substrate for CD45, referred to as pCAP-SP1, confirmed to be CD45 specific.^28^ When pCAP-SP1 is dephosphorylated by CD45, the resultant CAP-SP1 emits fluorescence which can be captured on a flow cytometer (Attune NxT) using a Pacific Blue emission filter. CAP-SP1 fluorescence correlate with CD45 activity and can be paired with staining for surface and intracellular markers. Note that CD45 activity and pCAP-SP1 are used synonymously throughout.

### Flow cytometry staining for surface and intracellular markers

Following the CD45 activity assay, dead cells were labeled using the Fixable Near-IR Dead Cell Stain Kit (Invitrogen) for 15 min at room temperature (RT). Afterward, antibodies directed against surface markers were diluted in fluorescence-activated cell sorting (FACS) wash and applied for 30 min on ice. Cells were washed to remove excess antibody and fixed/permeabilized using the Transcription Factor Buffer Set (BD Pharmingen) optimized for flow cytometric staining. Antibodies for intracellular targets were diluted in perm/wash buffer and applied for 40 min at 4°C. Excess antibody was removed, the cells were resuspended in FACS wash, and samples run on the flow cytometer.

### Flow cytometry antibodies

Antibodies (clone, company) used for flow cytometric staining at manufacturer recommend dilutions: CD4-Alexa Fluor 488 (OKT4, eBioscience), CD5-PerCP/Cyanine5.5 (L17F12, BioLegend), CD43-PE/Cy7 (CD4310G7, BioLegend), CD38-Alexa Fluor 700 (HB-7, BioLegend), galectin1-Alexa Fluor 647 (GAL1/1831, Novus Biologicals), Phospho Syk (Tyr348)-PE (moch1ct, eBioscience), Phospho BTK (Tyr223)-PE (A16128B; BioLegend), Phospho ERK1/2 (Thr202, Tyr204)-PerCP-eFluor 710 (MILAN8R; Invitrogen), Ki67-Brilliant Violet 711 (Ki-67, BioLegend), CD31-Brilliant Violet 711 (WM59, BioLegend), Human IgG PE (1268C, Bio-Techne), and CD38-FITC (T16, Beckman Coulter).

### Confocal microscopy

Isolated CLL cells were washed twice in PBS and gravity sedimented onto glass microscopy slides (SuperFrost Plus, Thermo Fisher Scientific) according to a protocol for attaching non-adherent cells.^55^ CLL cells were subsequently fixed with 4% PFA for 20 min at RT and washed three times with PBS. Incubations proceeded as previously described^24^ and images were acquired sequentially using an Olympus FV1000 confocal microscope. The following antibodies (clone, company, concentration) were resuspended in staining buffer (PBS with 2% BSA): Mouse anti-human CD45 (H130, BioLegend, 1:100), goat anti-human CD43 (polyclonal, R&D Systems, 1:50), rabbit anti-human CD38 (polyclonal, Thermo Fisher Scientific, 1:50), donkey anti-mouse Alexa Fluor 488 (1:500, Thermo Fisher Scientific), donkey anti-goat Alexa Fluor 555 (1:500, Thermo Fisher Scientific), and donkey anti-rabbit Alexa Fluor 647 (1:500, Thermo Fisher Scientific). All images were processed using the analysis software Fiji.^56^

The Duolink PLA (Sigma-Aldrich) was performed according to the manufacturer’s protocol. Cells were adhered, fixed, and washed as described in the above paragraph and imaged using a 100× UPlanFL objective. Mouse anti-human CD45 (HI30, BioLegend, 1:100) and rabbit anti-human CD43 (SP55, Abcam, 1:50) were used for the primary antibody incubations. Isotype controls, rabbit IgG (Invitrogen) and mouse IgG (ProSci), to confirm specificity of the PLA assay were included. Prior to analysis, brightness and contrast settings were adjusted and the same settings were applied to each image. Subsequently, image processing occurred as follows: Process-Subtract Background (50.0 pixels, sliding paraboloid); Process-Binary-Make Binary. Finally, the PLA signal was quantified using the Analyze Particle function (size 0.1–10 μm^2^) and cells identified based on DAPI staining.

### Quantitative real-time PCR

CLL cells and malignant B cell lines were harvested and RNA isolated with TRIZol reagent (Invitrogen). RNA concentration was measured using a DS-11 Series Spectrophotometer (DeNovix) prior to use of the TaqMan RNA-to-CT 1-Step Kit (Applied Biosystems) for reverse transcription and quantitative PCR. Samples were run on a StepOnePlus Real-Time PCR System (Applied Biosystems) with the following settings: reverse transcription at 48°C for 15 min, then 10 min at 95°C, followed by 40 cycles at 95°C for 15 s and 1 min at 60°C. All samples were run in triplicate with 10 ng RNA as templates per well in a 15-μL reaction volume with RNase-free water used in negative control wells. CD43 (*SPN*), galectin-1 (*LGALS1)*, and RNA polymerase II subunit A (*POLR2A*) TaqMan primer/probe sets not amplifying genomic DNA were purchased (Assay IDs Thermo Fisher Scientific: Hs01872322_s1, Hs00355202_m1, Hs00172187_m1). Quantification occurred via the comparative threshold cycle method (2^−ΔΔCT^ or 2^−ΔCT^) with normalization to the housekeeping gene *POLR2A*.

### Generation of CD38 and LGALS1 knockout cell lines

The Ramos cell line was employed to develop *CD38* and *LGALS1* knockout variants (Ramos.*CD38*^KO^ and Ramos.*LGALS1*^KO^, respectively) using CRISPR-Cas9 technology as previously described.^25^^,^^57^ Briefly, the online target site identification tool CHOPCHOP v3^58^ was used to design gRNA sequences (CD38.e4: 5′-GATCCTCGTCGTGGTGCTCG-3′, LGALS1.e3: 5′-AACCCTCGCTTCAACGCCCACGG -3′), which was inserted into single guide RNA expression cassette of the pSpCas9(BB)-2A-GFP vector (PX458, Addgene plasmid #48138). Ramos cells were electroporated using the Neon Transfection System (Invitrogen) and bulk sorted on GFP positivity 24 h after transfection. After expansion, single cell sorting was performed. Ramos.*CD38*^KO^ clones were screened and identified by flow cytometry. Ramos.*LGALS1*^KO^ clones were identified by quantitative real-time PCR.

### Statistical analysis

All statistical analyses were done using GraphPad Prism (GraphPad Software, Inc.). Datasets were assessed for a normal distribution using the D’Agostino & Pearson and Shapiro-Wilk tests. Data is presented as paired values or mean (parameter of interest) ± SD. Each pairing represents one patient sample. Statistical significance was defined as *p* values <0.05. Asterisks denote significance as such: ∗*p* ≤ 0.05; ∗∗*p* ≤ 0.01; ∗∗∗*p* ≤ 0.001; ∗∗∗∗*p* ≤ 0.0001. For two-way ANOVA analyses, black asterisks represent differences between Th versus CLL cells, red asterisks denote differences between CLL cells and control, and blue asterisks denote differences between Th cells and control.

## Data and code availability

For original data, please contact the corresponding author at johimb@ous-hf.no.

## Supplemental information

Document S1. Figures S1–S11 and Table S1

Document S2. Article plus supplemental information
