# Supplementary material for: CD38 regulates chronic lymphocytic leukemia proliferation via CD45 phosphatase activity
Source: Mol Ther Oncol. 2024 Jun 24;32(3):200841. doi: 10.1016/j.omton.2024.200841 (PMC11519781; doi:10.1016/j.omton.2024.200841)
Supplement: Document S1. Figures S1–S11 and Table S1 [file mmc1.pdf]

OMTON, Volume *32*

Supplemental information

CD38 regulates chronic lymphocytic leukemia proliferation via CD45 phosphatase activity

John F. Imbery, Celina Wiik, Julia Heinzelbecker, Jenny K. Jebsen, Mia K. Dobbing, Nunzio Bottini, Stephanie M. Stanford, Ludvig A. Munthe, Geir E. Tjønnfjord, Anders Tveita, Peter Szodoray, and Britt Nakken

Table S1: Patient Characteristics.
Age: Age at inclusion
IgVH: mutational status of immunoglobulin heavy chain. M=mutated UM=unmutated
CD38: Percentage of $CD38^{+}$ CLL cells compared to isotype control
NA: Not available

Patient Identifier	Gender/age (y)	IGVH (% germline)	CD38 (%)	Cytogenetic aberration
CLL 101	M/54	M (91.4) VH1-69	13	NA
CLL 106	F/56	M (95.2) VH3-23	1	NA
CLL 107	M/45	M (92.9) VH3-30	1	Heterozygous del(13q14)
CLL 116	M/68	M (93.4) VH4-59	2	NA
CLL 126	F/55	M (95.4) VH4-34	5	NA
CLL 142	F/37	M (96) VH3-07	2.5	Heterozygous del(17p13)
CLL 143	M/90	M biallelic (91, 88.7) VH3-33, VH4-59	<1	Heterozygous del(13q14)
CLL 145	M/53	M (93.1) VH1-2	1.0	NA
CLL 148	F/55	M (93.3) VH4-34	<1	NA
CLL 149	M/48	UM (100) VH3-15	1.7	NA
CLL 150	F/53	UM (100) VH3-33	13	Homozygous del(13q14)
CLL 151	F/51	M (93.2) VH3-7	3	NA
CLL 153	M/48	UM (100) VH3-48	49	Heterozygous del(11q22), trisomy 12
CLL 156	M/40	UM (100) VH4-34	43	Heterozygous del(11q22) No TP53 mutation
CLL 158	F/62	M (93.9) VH1-8	3	NA
CLL 163	F/61	M (96.9) VH3-9	1	NA
CLL 168	F/63	M (97.9) VH3-23	negative	NA
CLL 179	M/45	M (97%) VH4-39*01	22	46,XY; SF3B1 mutation (VAF 49%), no TP53 or NOTCH1 mutation
CLL 181	F/69	M (97.9) VH4-34	NA	46,XX,t(7;12)(q22;q24)
CLL 185	F/53	M (94.2) VH2-5	<1	NA
CLL 207	M/61	M (92.8) VH3-23	40	NA
CLL 222	F/43	M (97.3) VH2-5	5	NA
CLL 223	F/53	UM biallelic (100, 100) VH1-69, VH4-4	3	Trisomy 12 Homozygous del(13q14)
CLL 227	F/68	M (93) VH1-2	1	No TP53, NOTCH1, or SF3B1 mutation
CLL 239	M/64	M (92.4) VH2-5	1	NA

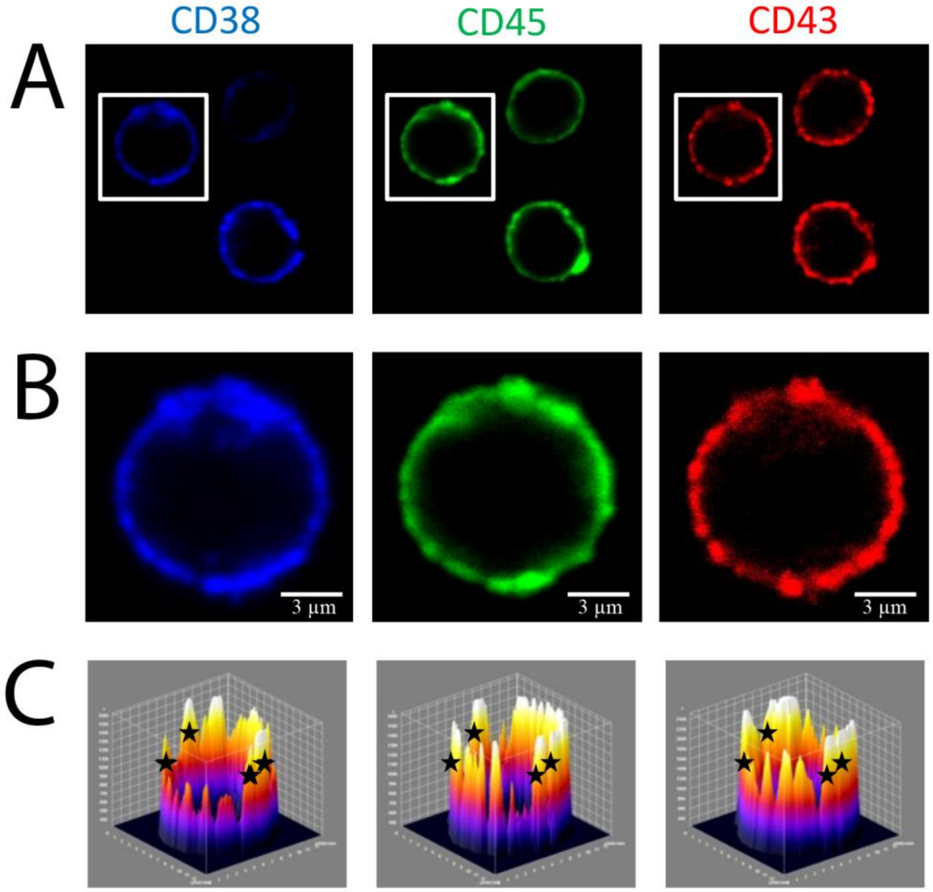

Figure S1. **3D projections of CLL cell CD38, CD45, and CD43 fluorophore pixel intensity.** The localization of CD38, CD45, and CD43 on the CLL cell plasma membrane was captured using immunofluorescence and confocal microscopy (row 1). From the outlined cell (row 2), a 3D projection of the fluorophore pixel intensity was generated for each protein's staining pattern using the Fiji—Analyze—3D Surface Plot function. In the 3D plots (row 3), similar areas of high intensity staining between the proteins are denoted by stars and the overlap suggests regions of tri-partite localization for the proteins of interest.

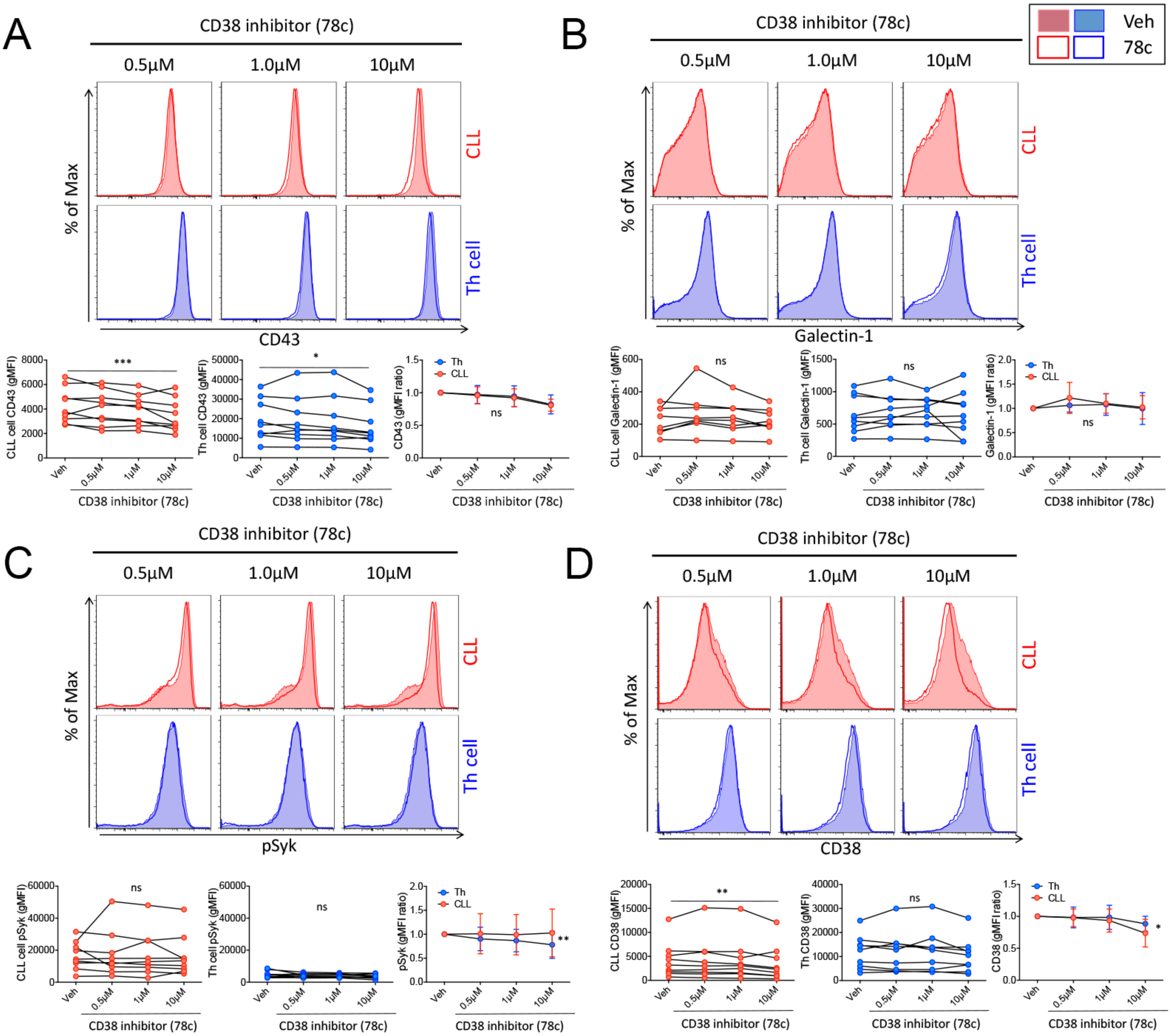

Figure S2. **CD38 enzymatic inhibition reduced expression of some CD45 activity regulators.** The surface expression of CD43 (A), Galectin-1 (B), and CD38 (D) was assessed alongside pSyk activation (C) in CLL and Th cells treated with CD38 enzymatic inhibitor 78c. Representative histograms are included from CLL (red, top rows) and Th cells (blue, bottom rows). Filled histograms represent vehicle treated cells, while non-filled histograms represent 78c treated cells. CD43 surface expression was reduced in CLL and Th cells (A), with a concomitant reduction in CD38 surface expression in CLL cells (D). No other significant results were observed (B, C). When comparing between Th and CLL cells, values have been normalized to vehicle (gMFI ratio). Statistical significance was defined by Friedman's test with Dunn's multiple comparisons test (B Galectin-1 CLL cells, C pSyk CLL cells, D CD38 CLL cells) or repeated-measures one-way ANOVA with Dunnett's multiple comparisons test for the remaining. When comparing between Th versus CLL cells, two-way ANOVA was used except for Galectin-1 where data was fitted with a mixed-effects analysis. Data is representative of 8 (CLL Galectin-1 gMFI) or 9 CLL patient samples.

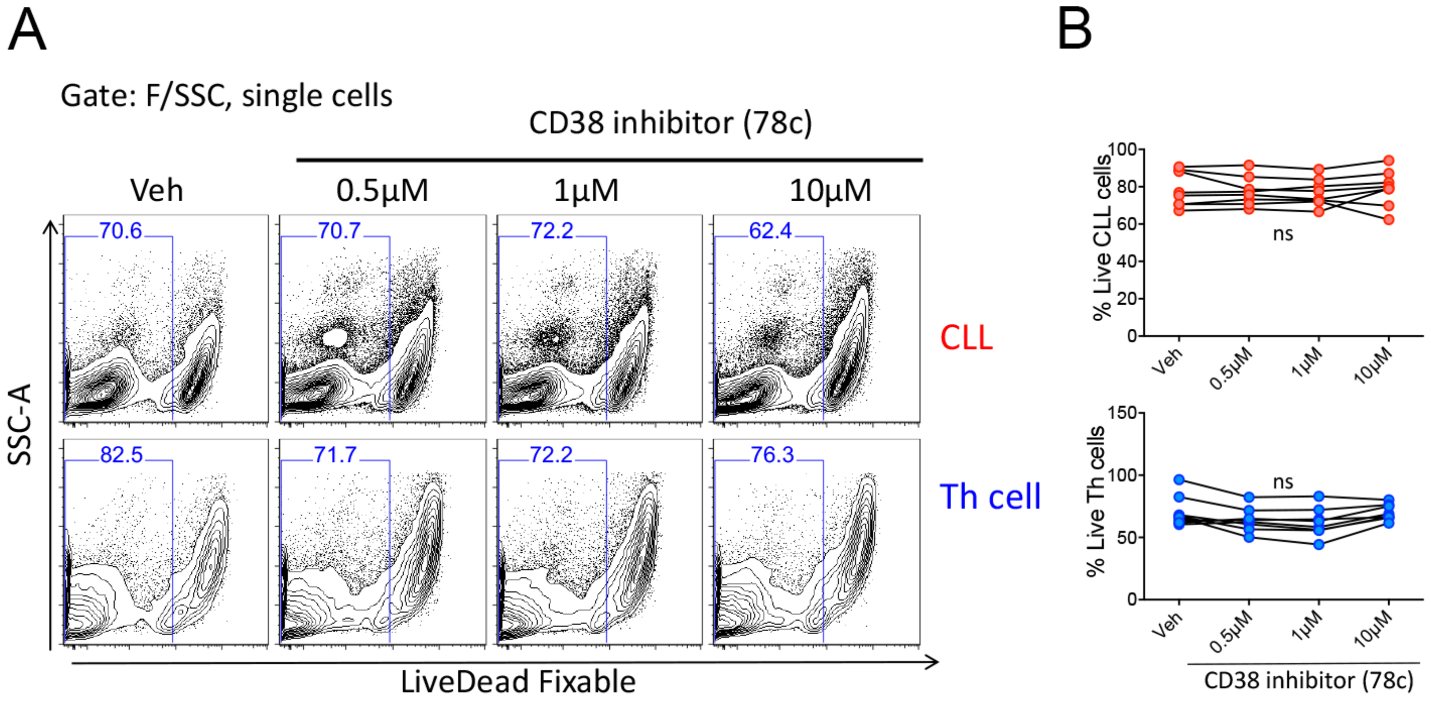

Figure S3. **CD38 enzymatic inhibition did not significantly alter cell viability.** (A, B) Use of enzymatic inhibitor 78c did not induce cell death in CLL (top row) or Th cell (bottom row) populations at the indicated concentrations. Included are representative flow cytometry scatter plots. Statistical significance was calculated by repeated-measures one-way ANOVA with Dunnett's multiple comparisons test. Data is representative of 8 CLL patient samples.

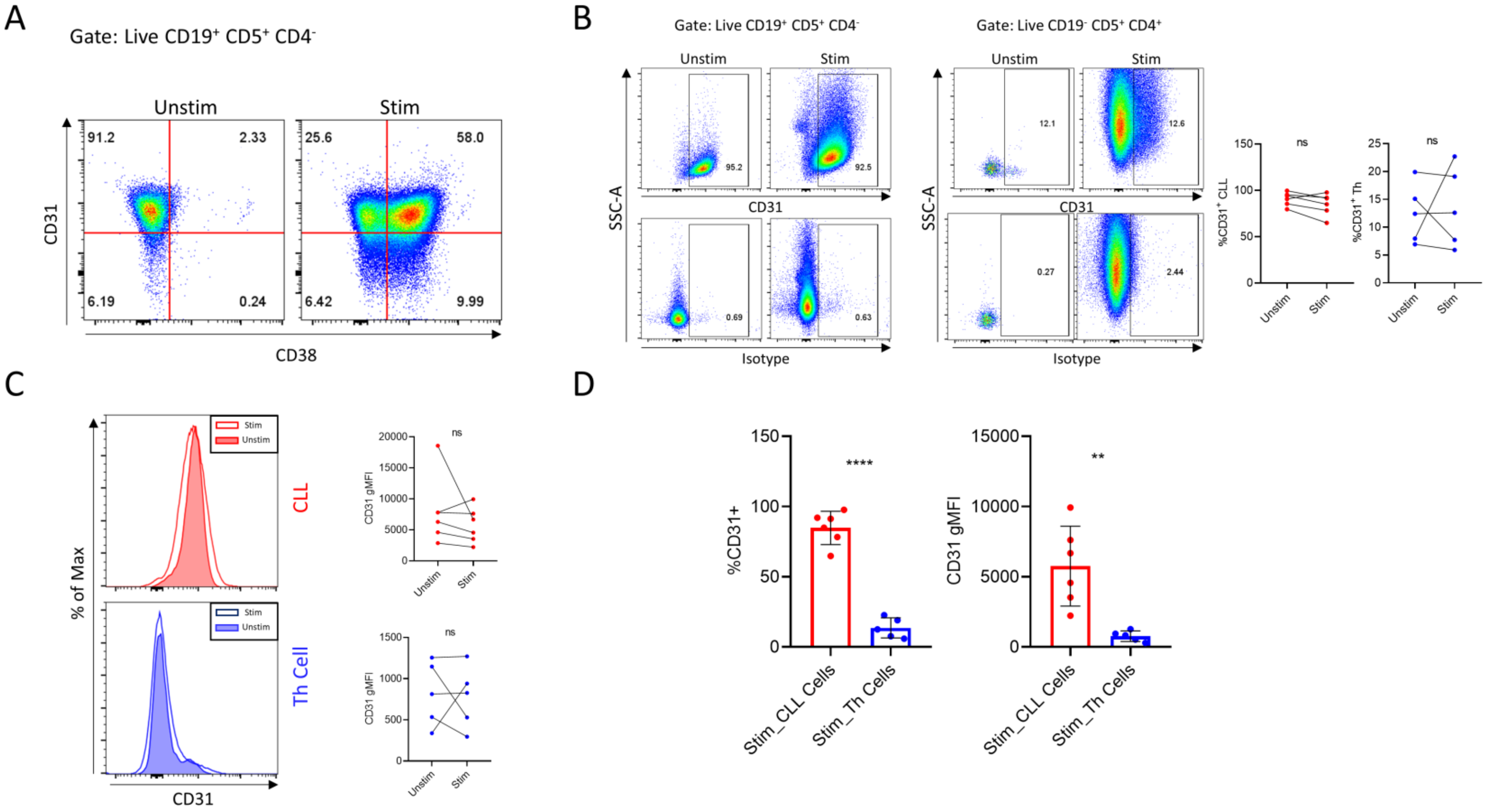

Figure S4. **The CD38 ligand CD31 is preferentially expressed on CLL cells and is unchanged by stimulation.** CLL and Th cells from the co-culture system were examined for CD31 expression. (A) Stimulated CLL cells co-express CD31 and CD38. Included are representative flow cytometry scatter plots. (B) The percentage of CLL and Th cells expressing CD31 remains unchanged between unstimulated and stimulated cultures. Included are representative flow cytometry scatter plots and their corresponding CD31 isotype control stain. (C) Overall CD31 expression (gMFI) also remained unchanged for CLL and Th cells when comparing between unstimulated and stimulated cultures. Included are representative histograms. (D) Stimulated CLL cells have a greater percentage of CD31 expressing cells and express more CD31 (gMFI) than stimulated Th cells. Statistical significance was calculated with Student's paired *t*-test (B), Student's ratio paired *t*-test (C), Student's unpaired *t*-test (D, %$CD31^{+}$), and Student's unpaired *t*-test with Welch's correction (D, CD31 gMFI). Data is representative of 5 (Th cells) and 6 (CLL cells) CLL patient samples.

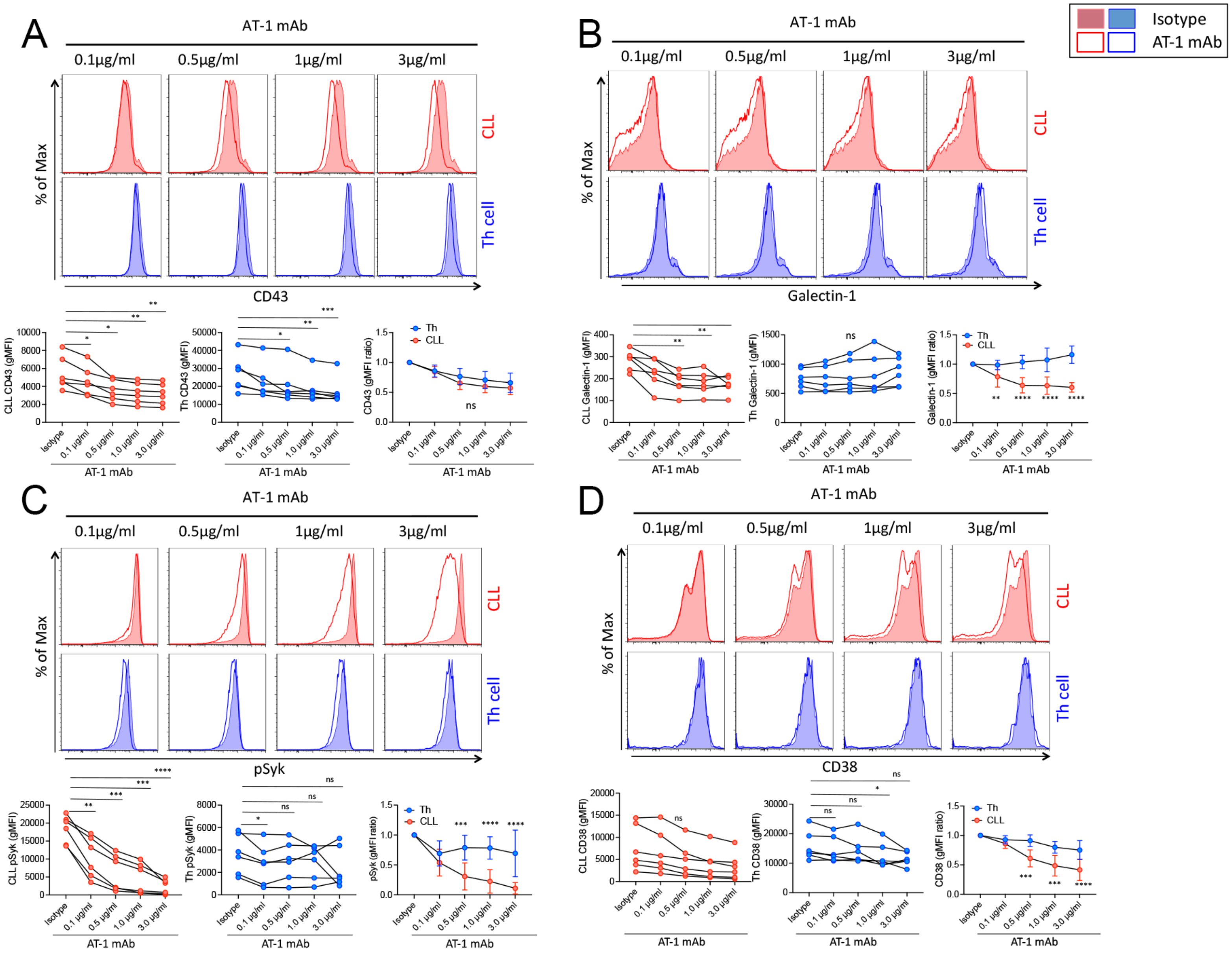

Figure S5. **CD38 receptor inhibition reduced pSyk activation and CD45 activity surface regulators.** Surface expression of CD45 activity regulators CD43 (A) and Galectin-1 (B) were assessed alongside pSyk activation (C) and CD38 expression (D) in CLL and Th cells following treatment with mAb AT-1. Top row histograms represent CLL cells (red), while bottom row histograms represent Th cells (blue). Filled histograms are isotype control and non-filled histograms are AT-1 treated. (A) CD43 surface expression was reduced both in Th and CLL cells at a similar rate. (B) Galectin-1 was selectively reduced in CLL rather than Th cells. (C) pSyk activation was reduced both in CLL and Th cells, and at a more robust rate in CLL cells. (D) Individual results displayed no significant trends. However, CLL CD38 expression was reduced when compared to Th cells. Th versus CLL cell graphs are normalized to isotype gMFI values (gMFI ratio). Statistical significance was calculated with Friedman’s test with Dunn’s multiple comparisons test (A for CD43 Th cells) or repeated-measures one-way ANOVA with Dunnett’s multiple comparisons test for the remaining. Two-way ANOVA was used for Th versus CLL cell comparisons. Data is representative of 6 CLL patient samples.

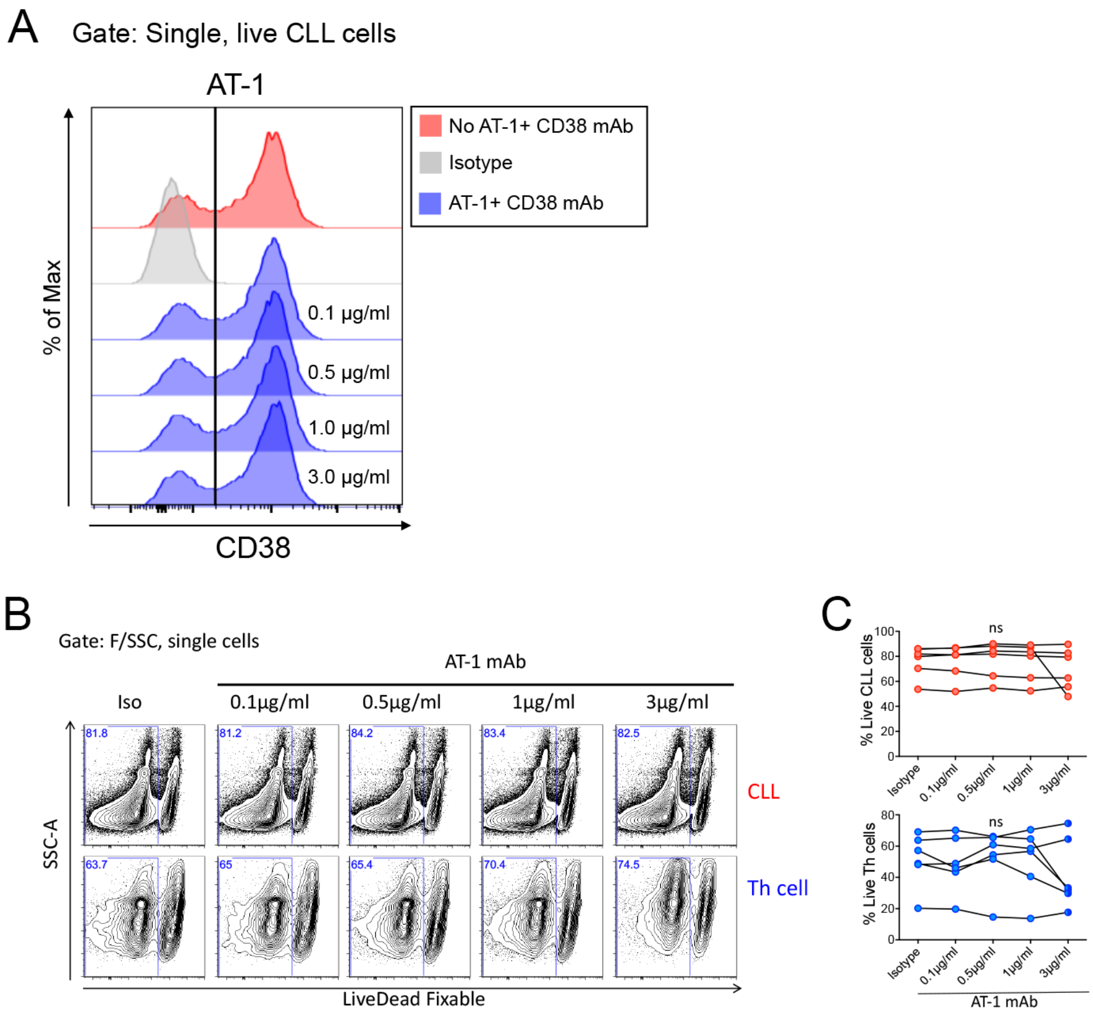

Figure S6. **CD38 receptor inhibition did not significantly alter cell viability or CD38 flow cytometer antibody staining.** (A) CLL cells were incubated on ice with or without AT-1 (to inhibit receptor-mediated endocytosis) and then stained with our CD38 flow cytometry antibody. AT-1 did not inhibit binding of our CD38 flow antibody at any experimental concentrations. Included are representative histograms. (B, C) Administration of the blocking CD38 mAb AT-1 did not significantly change the viability of CLL (top row) or Th cells (bottom row) at concentrations used in our experiments. Representative flow cytometry scatter plots are included. Statistical significance was determined by repeated-measures one-way ANOVA with Dunnett's multiple comparisons test. Data is representative of 6 CLL patient samples.

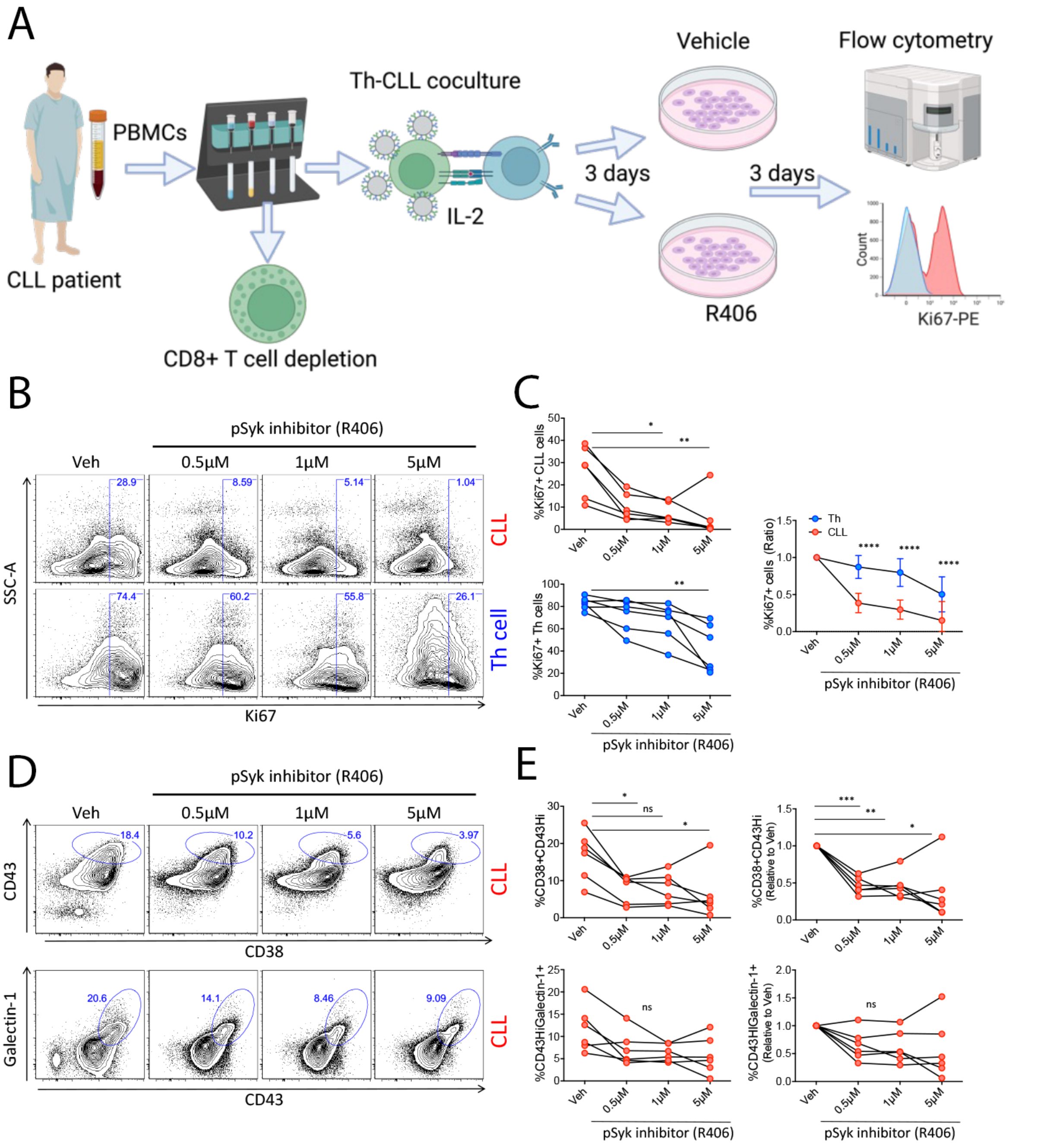

Figure S7. **Active pSyk kinase was instrumental for Th-cell mediated CLL proliferation and contributed to CD45 activity regulation.** (A) Schematic of the experimental setup. Created with BioRender.com. (B, C) Use of Syk inhibitor R406 robustly reduced CLL proliferation alongside a modest reduction in Th cell proliferation at the highest concentration of inhibitor. Comparison of Th versus CLL cell proliferative reduction showed a more significant effect in CLL cells. Representative flow cytometry scatter plots are included for CLL (top row) and Th cells (bottom row). %Ki67^{+} was normalized as ratio over vehicle. (D, E) R406 inhibition of Syk also down-modulated the CD45 activity^{hi} population CD38^{+}/CD43^{hi} (top row) but did not significantly affect the CD43^{hi}/Galectin-1^{+} population (bottom row). Included are representative flow cytometry scatter plots as well as graphs normalized as ratio over vehicle. Statistical significance was calculated by Friedman's test with Dunn's multiple comparisons test (C CLL cells, E %CD38^{+}/CD43^{hi} left graph), repeated-measures one-way ANOVA with Dunnett's multiple comparisons test (C Th cells, rest of E), and two-way ANOVA for the Th versus CLL cell comparison. Data is representative of 6 CLL patient samples.

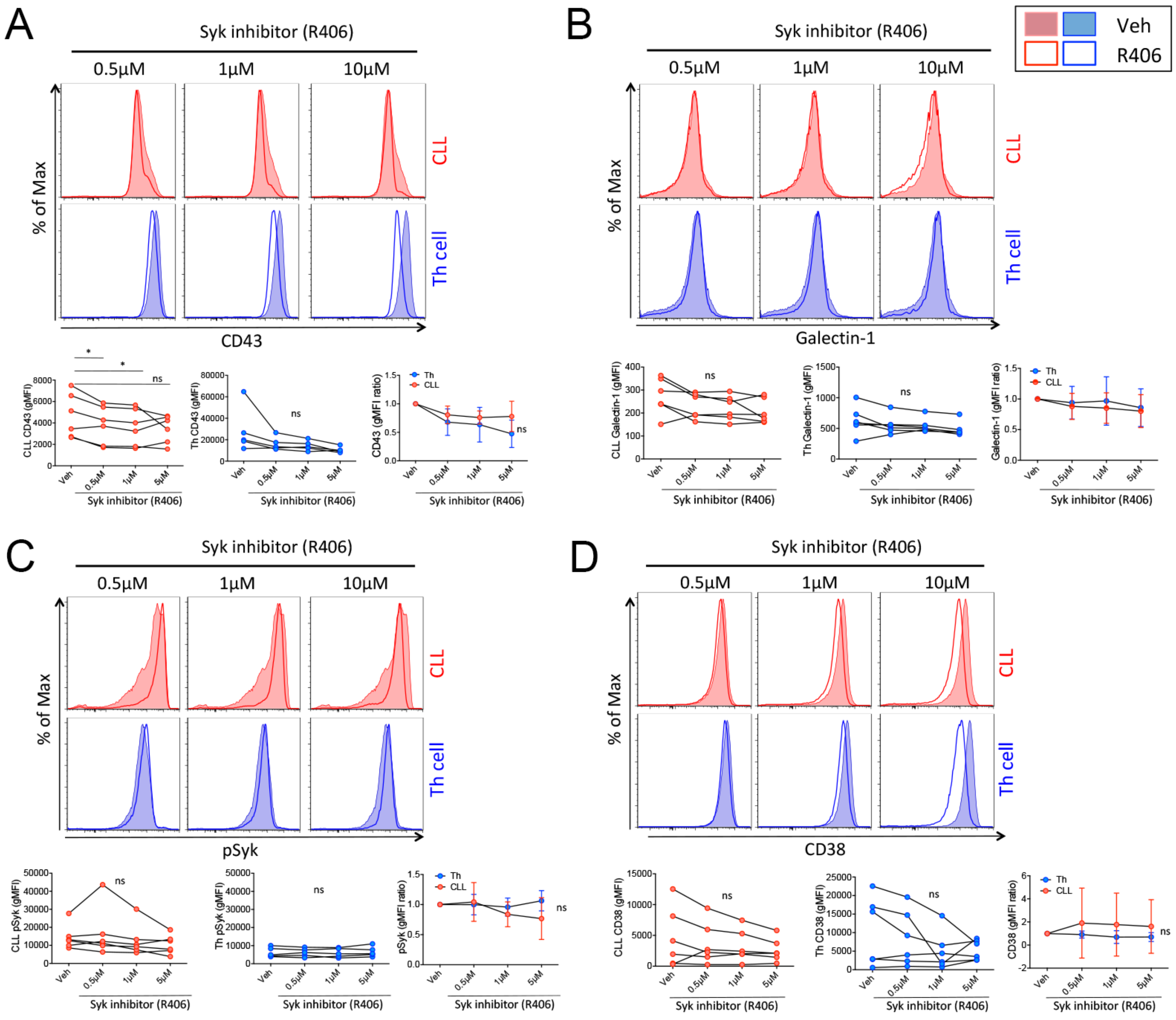

Figure S8. **Active Syk kinase contributed to regulation of CD43.** CLL and Th cells were evaluated for changes in expression of CD43 (A), Galectin-1 (B), pSyk activation (C), and CD38 (D) following use of Syk inhibitor R406. Representative histograms from CLL (top rows, red) and Th cells (bottom rows, blue) are included. Filled histograms are vehicle control and non-filled histograms are R406 treated. (A) CLL cells had a statistically significant reduction of CD43 expression at 1 µM of R406 but had a smaller yet non-significant reduction in CD43 when compared to Th cells. (B) Galectin-1 levels were decreased in 5 out of 6 patients for Th and CLL cells. (C) pSyk activation was inhibited in 5 out of 6 patients for CLL cells. (D) No changes or trends in CD38 expression were observed. In comparisons between Th and CLL cells, values were normalized to vehicle (gMFI ratio). Statistical significance was determined by Friedman's test with Dunn's multiple comparisons test (C pSyk CLL cells) or repeated-measures one-way ANOVA with Dunnett's multiple comparisons test for remaining CLL graphs. As R406 induced Th cell death at 5 µM in one patient, a missing value means a mixed-effects analysis with Dunnett's multiple comparisons test was used to analyze Th cell graphs. Similarly, a mixed-effects analysis with Sidak's multiple comparisons test was used for Th versus CLL analysis. Data is representative of 6 CLL patients.

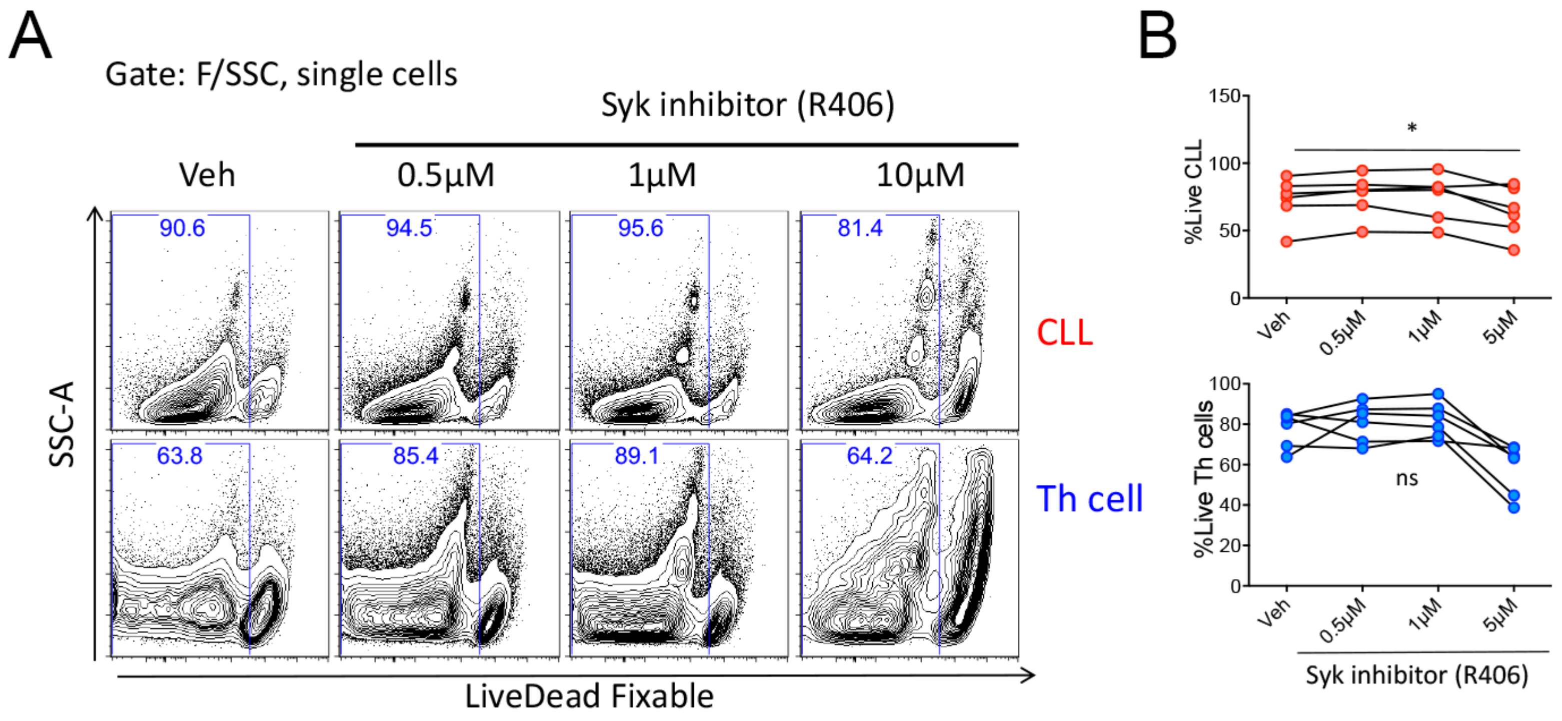

Figure S9. **Syk kinase inhibition moderately altered cell viability.** (A, B) Treatment with Syk inhibitor R406 had minimal effects on cell death in CLL (top row) and Th cell (bottom row) populations at the indicated concentrations. Representative flow cytometry scatter plots are included. Statistical significance was defined by repeated-measures one-way ANOVA with Dunnett's multiple comparisons test. Data is representative of 6 CLL patient samples.

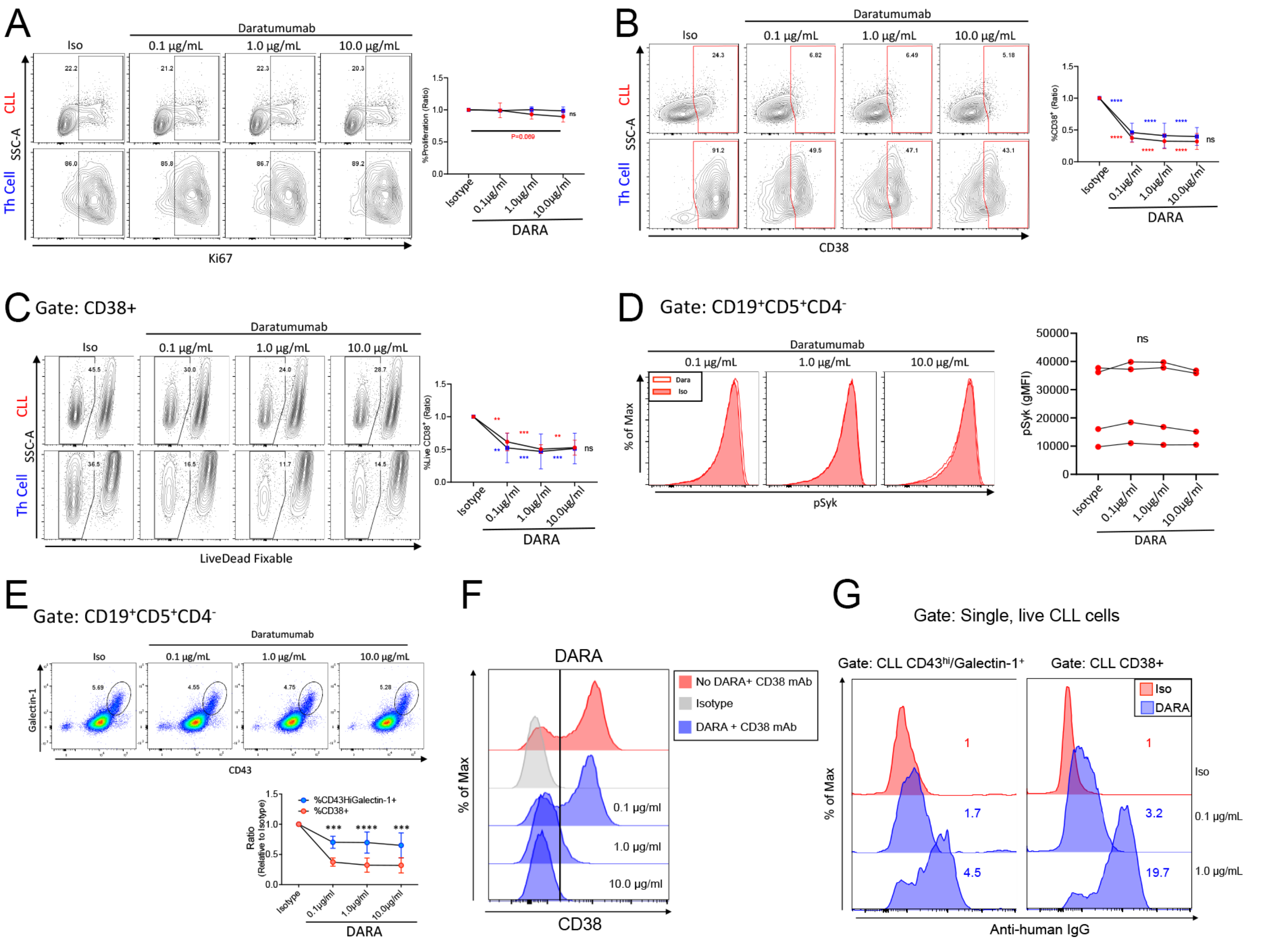

Figure S10. **Daratumumab reduced CD38$^+$ CLL cells in an autologous Th-CLL system.** (A) The proliferative (Ki67$^+$) output of CLL cells was modestly, though not significantly, reduced following treatment with daratumumab (DARA) at the highest tested concentration in comparison to isotype antibody control. Th cells remained unperturbed. Comparisons between the cell types demonstrated a slight trend where CLL cells displayed a greater proliferative reduction. Included are representative flow cytometry scatter plots (top row: CLL cells; bottom row: Th cells). (B) There was a significant reduction in %CD38$^+$ CLL and Th cells upon DARA treatment. Representative flow cytometry scatter plots are included (top row: CLL cells; bottom row: Th cells). (C) DARA induced cell death in CD38$^+$ CLL and Th cells at all tested concentrations and did so at a similar level in both cells types. Representative flow cytometry scatter plots are included (top row: CLL cells; bottom row: Th cells). (D) Assessment of pSyk activation as readout of tonic BCR signaling revealed no changes following administration of DARA at the tested concentrations. Representative histograms are included. (E) The CD43^{hi}/Galectin-1$^+$ population remained stable across the tested concentrations of DARA and did not reduce at a similar rate when compared to %CD38$^+$ CLL cells. (F) Cells were incubated on ice with or without DARA (to prevent receptor-mediated endocytosis) and then stained with our CD38 flow cytometry antibody. DARA prevented binding of our CD38 flow antibody only at the intermediate and high concentrations. (G) We also tested the ability of DARA to bind the CD43^{hi}/Galectin-1$^+$ and CD38$^+$ CLL populations by staining with an anti-human IgG antibody. Representative histograms are included. The values reference gMFI human IgG staining relative to isotype (non-DARA treated). Included are representative histograms. Statistical significance was determined by Two-way ANOVA (A-C, E) and repeated-measures one-way ANOVA with Dunnett's multiple comparisons test (D). Data is representative of 4 CLL patient samples.

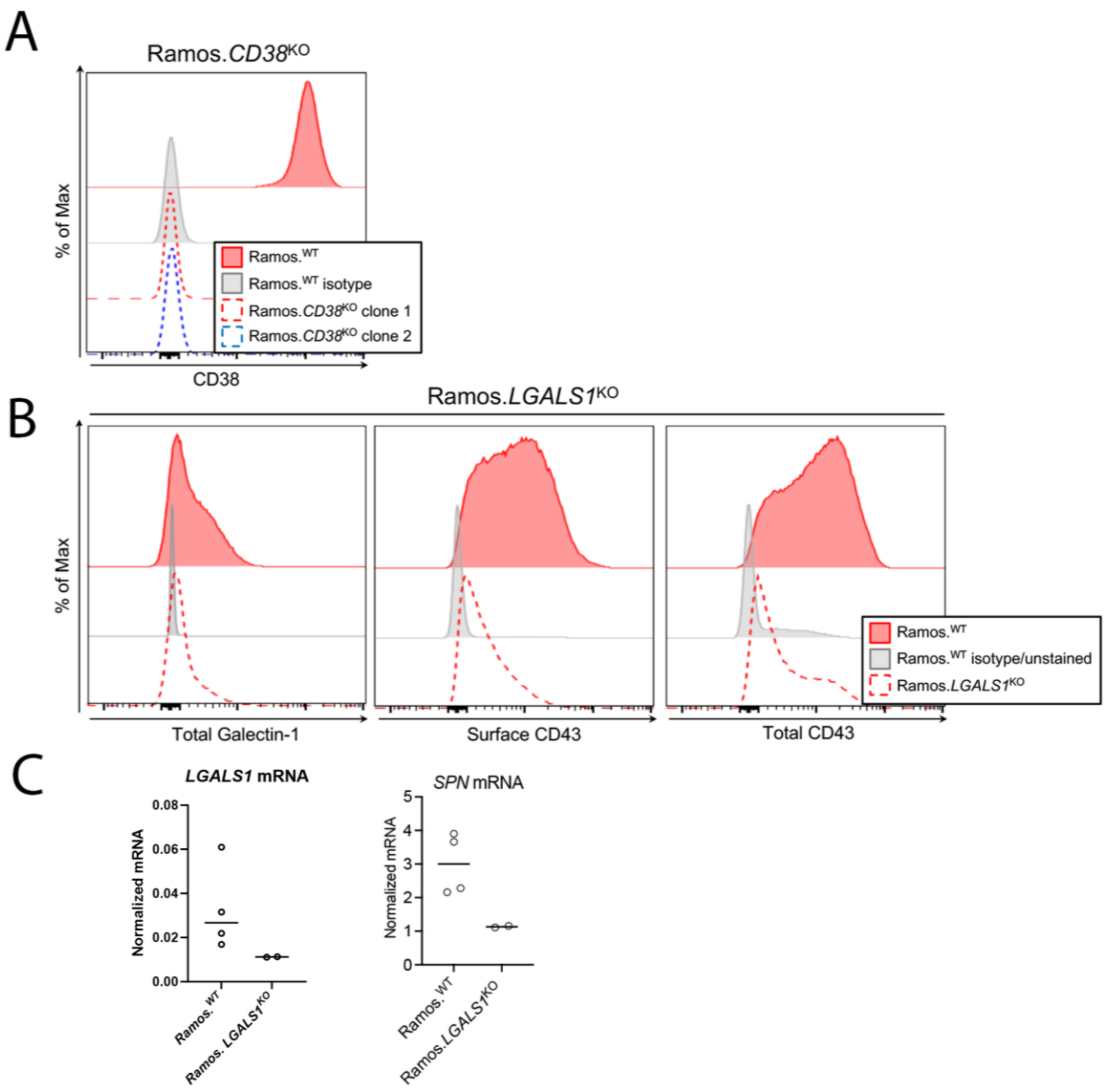

Figure S11. **Generation of Ramos *CD38*- and *LGALS1*- deficient cell lines.** CRISPR-Cas9 was used to ablate *CD38* or *LGALS1* gene expression in Ramos cells. (A) Knockout of CD38 was confirmed using flow cytometry and expression was reduced to isotype control levels in two separate clones. Included are representative histograms. (B) Flow cytometry was used to assess expression of total Galectin-1, surface CD43, and total CD43 in Ramos.^{WT} (red filled histograms) versus Ramos.*LGALS1*^{KO} cells (dotted red lines). Expression was reduced for each parameter. Gray filled histograms represent unstained (Galectin-1) or isotype (CD43) controls. (C) qRT-PCR analysis of *LGALS1* and *SPN* (CD43) mRNA in Ramos.^{WT} and Ramos.*LGALS1*^{KO} cells.
